# Does observability affect prosociality?

**DOI:** 10.1098/rspb.2018.0116

**Published:** 2018-03-28

**Authors:** Alex Bradley, Claire Lawrence, Eamonn Ferguson

**Affiliations:** Personality, Social Psychology, and Health (PSPH) Research Group, School of Psychology, University of Nottingham, Nottingham NG7 2RD, UK

**Keywords:** visibility, prosocial, cost signalling theory, indirect reciprocity, competitive altruism

## Abstract

The observation of behaviour is a key theoretical parameter underlying a number of models of prosociality. However, the empirical findings showing the effect of observability on prosociality are mixed. In this meta-analysis, we explore the boundary conditions that may account for this variability, by exploring key theoretical and methodological moderators of this link. We identified 117 papers yielding 134 study level effects (total *n* = 788 164) and found a small but statistically significant, positive association between observability and prosociality (*r* = 0.141, 95% confidence interval = 0.106, 0.175). Moderator analysis showed that observability produced stronger effects on prosociality: (i) in the presence of passive observers (i.e. people whose role was to only observe participants) versus perceptions of being watched, (ii) when participants’ decisions were consequential (versus non-consequential), (iii) when the studies were performed in the laboratory (as opposed to in the field/online), (iv) when the studies used repeated measures (instead of single games), and (v) when the studies involved social dilemmas (instead of bargaining games). These effects show the conditions under which observability effects on prosociality will be maximally observed. We describe the theoretical and practical significance of these results.

## 1. Introduction

There are a number of possible ultimate-proximate accounts for why altruism survives in the population [1–3]. For example, kin selection and inclusive fitness offer an ultimate functional account of altruism that is enacted, at a proximate level, by mechanisms of kin detection [4,5]. Here, we explore functional level theories of altruism/prosociality that focus on the capacity of altruistic acts to signal desirable traits and qualities of the signaller (costly signalling theory, reciprocity and completive altruism). These desirable altruistic traits may provide a fitness advantage by being sexually selected [6] and/or increase the probability that the signaller will be helped in the future [7]. Specifically, we focus on one key component of these functional accounts: the link between the observability of a prosocial act and the degree of prosociality displayed. We also explore the moderating effects of key theoretical mechanisms (e.g. possibility for repeat interactions).

A number of functional theoretical accounts propose that the survival of prosocial behaviour depends on the signalling of desirable traits. This is premised on these traits being observable. For example, cost signalling theory (CST) suggests that observable acts signal the actor's qualities to potential mates [8]. Furthermore, for reciprocity (either direct or indirect) to be effective in maintaining prosociality, others need to be aware of the prosocial behaviour either directly (e.g. directly observed) or indirectly (e.g. via gossip) [9]. However, the empirical literature, based on both laboratory and the field studies, offers mixed findings, with evidence that observability increases [10,11], decreases [12] or has no effect [13,14] on prosociality. We propose that this variability reflects the presence or the absence of key theoretical design features, or mechanisms, that are required for the effect of observability on prosociality to emerge. These theoretical design features mark the *boundary conditions* for the emergence of observability–prosociality effects. For example, displays of prosociality that have a consequential impact on the helper should lead to increased levels of prosocial behaviour. If the mechanism of consequentiality is not part of a study's design, effects of observability will be weaker or non-existent. We examine this as one of many theoretically mechanisms that should moderate the observability–prosociality link.

While the literature on one aspect of observability—the presence of images of ‘watching eyes’—on prosociality has been reviewed [15–17], there is no systematic review of the effect that observability, in general, has on prosociality. Thus, a systematic review and meta-analysis of this literature to identify the boundary conditions for the observability–prosociality link is important for two reasons. First, given the importance of observability to theories of prosociality, a more nuanced understanding of boundary conditions will enhance our theoretical understandings. Second, while a number of charities use observability as a means to enhance revenues [10], the evidence is not clear as to how effective this is, or what features of manipulations positively impact fundraising.

### (a) Theoretical accounts of observability and prosociality

Below we outline the three main functional accounts of prosociality that rely on observability of prosocial behaviours: CST [8,18], competitive altruism [19,20] and reciprocity [9].

CST suggests that observability of costly prosocial acts is used to advertise to others their qualities as a potential partner or member of their group [18]. Competitive altruism suggests that human competitiveness influences prosocial displays, with people competing to gain a good reputation through displays of their prosocial qualities [21,22].

With respect to reciprocity, direct and indirect reciprocity can be distinguished [23]. Direct reciprocity occurs where an individual (A) performs a generous act to help another (B) and B subsequently repays A (A helps B, B then helps A) [9]. Indirect reciprocity comes in two forms: upstream and downstream [9]. In upstream reciprocity, an individual who has received help goes on to help others (A helps B, then B helps C), whereas downstream reciprocity occurs when a person who has helped others in the past has a higher likelihood of being helped by others in the future (A helps B, then C helps A) [19]. Upstream reciprocity is driven by the psychological mechanism of gratitude (B is grateful to A, so helps C) and downstream reciprocity by reputation building (B gains reputation from helping A, so is helped by C) [19,24]. For both direct reciprocity and downstream indirect reciprocity, reputation is the most likely mechanism operating and this requires that the prosocial act is observable either directly or via gossip.

The consistent prediction of these theories is that the effect of observability should be strongest, or only apparent, when the display of prosociality increases the helpers’ reputation, status or attractiveness and helps them to attain positive future benefits. Although we do acknowledge that in specific contexts, reputations for being prosocial could be taken advantage of [25,26], in the majority of situations it would yield positive future rewards [27,28]. Thus, the observability–prosociality link should have clear boundary conditions that maximize the chances for observability to enhance reputation, status or attractiveness. In the meta-analysis, we test this prediction directly and a number of other predictions (detailed below).

### (b) Defining prosocial behaviour, observability and different types of observation

Prosocial behaviour refers to a broad range of behaviours, efforts or intentions designed to benefit the welfare of individuals, groups, organizations or society [20,24]. Previous researchers have suggested that observability relates to the visibility of individuals' behaviours [29]. As a working definition, we suggest that the level of observability depends upon: (i) what information is revealed about an individual's identity (i.e. name and facial photograph), and (ii) what is revealed about that individual's behaviour (i.e. whether they made a donation or how much they donated). Using these two criteria, observability manipulations within the literature can be categorized at the point of the prosocial decision into one of three categories: perceived, pseudo and overt observability. Perceived observability refers to situations where actual information about both individual's identity and their prosocial behaviour is not observable. For example, the presence of images of ‘watching eyes’ creates a subjective perception that someone is watching the individual with no actual transmission of identity or information about their behaviour [11]. Pseudo-observability refers to situations where information about an individual's behaviour is observed (i.e. how much they donated), but no information about their actual identity is revealed at the point of the prosocial act. Instead, indirect identity information is revealed such as a participant identification (ID) number. While the ID number may be used to track whom people are playing with over rounds, and how much they have donated, it does preclude people being able to infer the person's age or sex, for example. The participant also knows what information others know about them. For example, studies that compare single-blind conditions where an experimenter knows the participant's ID number and donation amount to double-blind control conditions [30]. Finally, overt observability relates to manipulations where both actual identity information (e.g. name and face) and donating behaviour are revealed to an audience at or after the point of decision and this is known to the participant before their decision is made. For example, [31] used a public goods game where each player had their name, photograph and contribution displayed to all other players in the game.

### (c) Moderators of observability

We outline predictions for five theoretical mechanisms that can moderate the observability and prosociality relationship (e.g. decisions with consequences), as well as, examining several methodological moderators.

### (d) Theoretical moderators

#### (i) Type of observability manipulation

All theories (CST, competitive altruism and reciprocity models) predict that overt manipulations of observability, where there is the strongest opportunity to build a reputation, should lead to the highest levels of prosociality.

#### (ii) Observer type

We categorize observers into four distinct levels: (i) no one is observing (includes perceived manipulations), (ii) only an experimenter is observing, (iii) peers are watching (other participants performing the experiment), and (iv) passive observers are watching (individuals who are observing but are not participants or experimenters). An important feature of reciprocity is that others have the ability to recompense a victim or punish a transgressor [32,33]. Thus, we would expect that peers will have more of an effect on prosociality than having an experimenter watching, a passive observer, or having no observers (e.g. in the case of ‘watching eyes’).

#### (iii) Decisions with consequences

We distinguish between consequence-free decisions and decisions with consequences. Consequence-free decisions are when the actor performs a behaviour (e.g. a financial allocation) expecting their behaviour not to influence how others will respond to them within the experimental protocol. Decisions with consequences occur where actors expect their behaviour to influence how others will respond towards them within the experimental protocol. Again all theories (CST, competitive altruism and reciprocity) predict that effects of observability will be maximal with consequential decisions.

#### (iv) Single or repeated interactions

Repeated interactions with the same person/people facilitate reputation building [34]. Thus, reciprocity theory would predict that repeated tasks involving multiple interactions with the same individuals should lead to more prosocial behaviour if the acts are observable.

#### (v) Bargaining and social dilemma games

Social dilemmas are played with a group of people (e.g. public goods games usually have four players), whereas bargaining games tend to be played between two people (e.g. dictator games). The more players within the game, the larger the audience for the observed prosocial behaviour and the increased potential for reputation building [35]. In addition, the structures of the two types of games vary. Social dilemmas involve a trade-off between short-term interest and the long-term collective interests, and bargaining games involve a trade-off between personal outcomes and the outcomes of others [36]. Thus, social dilemmas involve long-term consideration about one's contributions to a common resource and the opportunity to enhance one's reputation within a group. Bargaining games encourage more short-term profit-maximizing behaviour and offer less opportunity for reputation building. Thus, we predict a stronger effect of observability on prosociality in social dilemmas owing to the larger audience and the longer reputation management that is needed.

### (e) Methodological moderators

These moderators are not mechanisms *per se*, but rather reflect experimental design choices that may influence the observability–prosociality link.

#### (i) Age

There may be developmental trends on the impact of observability on prosociality; therefore, we include age as a continuous covariate. Evidence within children (less than 12 years old) for observability effects on prosocial behaviour is mixed with some studies finding observable scenarios increase prosocial behaviour [37,38], while others find no effect [39]. We make no specific prediction about the effect of observability on prosociality in children.

#### (ii) Type of payment

Payment of participants can be done using a variety of methods, for example, raffles, conditional lotteries and flat fees. We categorized payments into three broad categories: no payment, one-off payment and performance-related pay (i.e. payment is conditional upon participant's decisions/behaviour). All theories would suggest that performance-related payments ought to lead to high prosocial behaviour under observability because it provides a clearer signal of generosity.

#### (iii) The context of study

A number of papers suggest that the level of prosociality in the field is lower than in the laboratory [40,41]. Therefore, the context of the study might act to moderate the observability and prosociality relationship with field studies having smaller effects than laboratory studies.

#### (iv) Nature of outcome

When analysing prosocial behaviour, researchers examine the decision to give or not (yes versus no) and how much to give, when giving is chosen [42]. Nettle *et al*.'s [16] meta-analysis on the ‘watching eyes effect’ on prosocial behaviour found that while the presence of images of ‘watching eyes’ seemed to affect the likelihood of donating (yes versus no decisions), it did not affect the amount donated. We explore whether this effect is observed in the wider observability literature.

#### (v) Unearned versus earned endowments

A number of studies show that when participants earn money during an experiment, as opposed to just simply receiving money (endowment/windfall), levels of generosity decrease [43]. As earned money entails property rights, donations signal a cost to that individual which could constitute an honest signal [35]. Thus, we predict that effects of observability will be greater for earned compared with unearned endowments.

#### (vi) Measures of outcome

We investigate whether the measurement of prosocial behaviour involved objective measures (e.g. expenditure of money) or subjective measures (e.g. self- or peer-reported intentions to perform a prosocial behaviour). The intention-behaviour gap is well established [44], and intentions to help can be a cost-free means to enhance reputation. Therefore, we expect to see stronger observability effects on prosocial behaviour in studies assessing self-reported intentions than studies assessing behaviour. That is people will be more likely to say they will help when it does not cost them, especially when observed.

#### (vii) Type of prosocial behaviour

The effects of observability have been researched over a variety of different prosocial activities: blood donation [10], donations to charity [10], donation to peers [13], volunteering time to charities [45,46], performing effortful tasks to raise money for charity [47], and voting [14]. All models predict, to different extents, that social recognition occurs in proportion to the cost of generosity signalled [46]. It could be argued that non-monetary forms of prosocial behaviour signal greater cost with respect to social impact (time and effort) because, at the point of donation, the amount of time given or blood that is donated incurs a known effortful cost, while the origin of the money being donated (windfall or effort fully earned) is less transparent. Thus, while someone may incur effort and time to gain money, this effort is not necessarily specifically linked to prosociality—it may just be their everyday work. At the point of donation, donating money is relatively less effortful and time-consuming than volunteering or donating blood. Thus, we predict that observability will show a stronger effect on non-monetary prosocial acts compared with monetary ones.

## 2. Method

### (a) Search strategy

Studies were identified through searching electronic databases, backward citation searches and contacting authors for unpublished data. Five electronic databases were searched: Web of Science, Psychinfo, Econ Papers, Taylor and Francis and the Applied Social Science Index and Abstracts (ASSIA). Searches comprised the combination of seven terms for observability (observability, visibility, public, reputation, signalling, anonymity, image) and seven terms for prosociality (money, blood, volunteer, recycle, altruism, prosocial, public good). All searches were conducted from 25 November 2015 to 31 March 2017. A total of 11 339 articles were searched via titles and abstracts. Of the 11 339 articles, 114 were identified as being potentially relevant. Backward citation searching through the 117 records led to another 55 papers being identified (number of papers: 169). An email was then sent to first authors (*n* = 61) requesting any unpublished data which produced further 18 papers (total number of papers: 187). A reviewer recommended a further 15 papers (total number of papers: 202). The full text of the remaining 202 articles was inspected and 83 articles that did not fulfil our eligibility criteria (see below) were excluded (see the electronic supplementary material, S5 PRISMA Flow Diagram, electronic supplementary material, figure S2).

### (b) Inclusion and exclusion strategy

The following inclusion and exclusion criteria were applied. First, studies had to include: (i) an observability manipulation (where the extent of participants actions and/or identity have been revealed, to some degree, to others whether actual or perceived), (ii) a control group (a group that received no manipulation or received manipulation that did not affect their observability), and (iii) a measure of prosocial behaviour (intentions or behaviour that benefited either an individual, group, organization or society) (67 out of 202 records did not match these criteria). A number of papers, especially those looking at indirect reciprocity, appear relevant to include but fail to provide a control group or visibility manipulation hence cannot be used to generate effect sizes (i.e. [48–51]). For example, in Milinski *et al*. [7], all players in the indirect reciprocity game have their decision to give or keep publically displayed each round (no control group) or in Bó [51] all players playing the Prisoner Dilemma games are anonymous to other players (no manipulation of visibility). Second, papers had to be written in English. Third, studies had to provide primary quantitative data (i.e. theoretical paper, simulations or qualitative papers were not included) (nine records did not match this criterion). We did not impose age restrictions on papers in the present review but included age as a continuous moderating variable instead. Fourth, no restrictions were placed on the years they were published or on publication type (i.e. working papers, theses were also included). Finally, papers were searched to remove duplicates (seven records were removed) and requests were sent to authors when not enough information to compute effect sizes were reported (two records were removed).

After applying all the inclusion and exclusion criteria, there were 117 papers with a total sample of 788 164 and 134 independent study level effects (independent effects that represent a study).

### (c) Coding scheme

See the electronic supplementary material S6 Coding Scheme (electronic supplementary material, table S4) for details of the coding framework.

### (d) Overview of the analysis

The effect sizes computed represent the difference between a control condition and an observability manipulation. Fischer's Zr was used for all the analyses, but for ease of interpretation, these are converted back to Pearson's *r* when reporting effects. The correlation coefficient r was computed using sample sizes and *t*-values, *χ*^2^-values, *p*-values, means and standard deviations, as well as, 2 × 2 contingency tables. Effect sizes are all scored such that the higher the correlation, the stronger the effect of observability on prosociality.

A random-effects model was used to compute the overall effect size of observability on prosociality (for justification of model choice, see the electronic supplementary material S7 Overview of Analysis). To handle non-independence, effect sizes were aggregated using the Hunter & Schmidt [52] method which combines within-study effects while taking account of the correlation in the within-study effects [52]. Effect sizes that were independent received no corrections.

Cochran's *Q*-test, a measure of homogeneity among effect sizes, was conducted to test whether the assumption that all effect sizes are estimating the same population mean is reasonable. To measure the degree of heterogeneity, the inconsistency index (*I*^2^) and tau squared (*τ*^2^) were calculated (For details, see the electronic supplementary material S7 Overview of Analysis).

Publication bias was assessed visually with a funnel plot (effect size plotted against the standard error) and statistically using Egger's Regression test. The Duval and Tweedie [53] trim and fill procedure was also applied (For details, see the electronic supplementary material S7 Overview of Analysis).

To explore heterogeneity, mixed-effect models are conducted using both theoretical and methodological moderators. To assess the overall effect of multiple moderators within mixed-effect models, omnibus tests of all the model coefficients were conducted (referred to as QM). Effect sizes were calculated using comprehensive meta-analysis [54], while all other analyses were conducted in R Studio (v. 0.98.1062) using R (v. 3.1.1) with the MAc and Metafor packages [55,56].

### (e) Coding frame reliability

The reliability of the coding framework was tested on 32% of studies (*k* = 43) by a rater who was blind to the initial coding (see the electronic supplementary material, S7 Overview of Analysis). The kappa coefficients indicate substantial agreement (mean kappa = 0.86, s.d. = 0.13, min = 0.67, max = 1.00) [57].

## 3. Results

### (a) Overall effects of observability on prosocial behaviour

An overview of the effect sizes and 95% confidence intervals for each study can be found in the electronic supplementary material, S1 Overview of Studies (electronic supplementary material, table S1).

The analysis revealed a small positive and significant association between observability and prosociality *r* (*r* = 0.141, 95% confidence interval lower limit (LLCI)/upper limit (ULCI) = 0.101/0.175). The results showed a substantial level of variation (*Q* (133) = 10883.758, *p* < 0.001) among the distribution of effect sizes which can be accounted for by the large amount of heterogeneity within the sampled studies (*I*^2^ = 99.24; *τ* = 0.179; *τ*^2^ = 0.032). ‘Leave one out’ sensitivity analysis was performed, where the model is run systematically leaving out one study each time. This showed the overall effect fluctuated slightly from *r* = 0.135 to *r* = 0.144, but to be stable. Trim and fill analysis and Egger's regression test indicated no publication bias (see the electronic supplementary material, S2 Publication Bias and figure S1).

### (b) Moderators of observability

In this section, we explore theoretical and methodological moderators that might account for some of the heterogeneity (see the electronic supplementary material, S3 Univariate Moderator Analyses and table S2).

#### (i) Theoretical moderators

*Type of observability manipulation.* While the effect of observability on prosociality was largest for overt manipulations (*r* = 0.172, LLCI/ULCI = 0.113/0.232, *k* = 60) followed by pseudo-observability (*r* = 0.167, LLCI/ULCI = 0.081/0.252, *k* = 20) and perceived observability (*r* = 0.100, LLCI/ULCI = 0.050/0.149, *k* = 49) manipulations, there was no overall significant difference across observability manipulations (QM (2) = 3.572, *p* = 0.167).

*Observer type.* Observer type was split into three dummy coded variables using no observer (perceived manipulations) as reference: experimenter, peers and passive observer. Observer type significantly moderated observability and prosocial behaviour (QM (3) = 13.246, *p* = 0.004) with passive observers (*r* = 0.352, LLCI/ULCI = 0.225/0.478, *k* = 12) having a significantly larger positive effect on prosociality than no observer (*r* = 0.100, LLCI/ULCI = 0.050/0.149, *k* = 49). All other observer types were not significantly different from having no observers (experimenter: *r* = 0.200, LLCI/ULCI = 0.090/0.309, *k* = 12; peers: *r* = 0.154, LLCI/ULCI = 0.085/0.224, *k* = 45). Passive observers also had a significantly larger effect on prosociality than peers (QM (1) = 7.30, *p* = 0.007). No other comparisons were significantly different.

*Decisions with consequences.* Decisions with consequences had a significantly (QM (1) = 7.365, *p* = 0.007) larger effect on the link between observability and prosociality than those with no consequences (decisions with consequences: *r* = 0.248, LLCI/ULCI = 0.148/0.348, *k* = 24; consequence-free: *r* = 0.119, LLCI/ULCI = 0.084/0.154, *k* = 110).

*Single or repeated measures.* Single versus repeated measure significantly moderated the link between observability and prosociality (QM (1) = 12.44, *p* < 0.001), with single studies having smaller effects (*r* = 0.106, LLCI/ULCI = 0.070/0.143, *k* = 94) than studies using repeated measures (repeated: *r* = 0.269, LLCI/ULCI = 0.179/0.358, *k* = 25).

*Bargaining games and social dilemmas.* The type of economic game (bargaining versus social dilemma) had a significant moderating effect (QM (1) = 6.963, *p* = 0.008), with social dilemmas having a larger effect (bargaining games: *r* = 0.119, LLCI/ULCI = 0.072/0.165, *k* = 74; social dilemma games: *r* = 0.251, LLCI/ULCI = 0.157/0.344, *k* = 26).

#### (ii) Methodological moderators

*Age.* The continuous variable of age had no moderating effect (QM (1) = 0.219, *p* = 0.639) on the observability and prosociality relationship (*r* = -0.001, LLCI/ULCI = -0.005/0.003, *k* = 65).

*Type of payment.* While one-off payments had the smallest effect on the link between observability and prosocial behaviour (*r* = 0.080, LLCI/ULCI = -0.021/0.181, *k* = 18) followed by no payment (*r* = 0.139, LLCI/ULCI = 0.085/0.194, *k* = 18) and performance-related payment (*r* = 0.157, LLCI/ULCI = 0.106/0.208, *k* = 80), there was no significant overall difference across payment types (QM (2) = 1.896, *p* = 0.388). There was also no difference between one-off payments and performance-related payments (QM (1) = 1.816, *p* = 0.178).

*Context of the study.* The study context (laboratory versus field) had a moderating influence on the observability–prosociality link (QM (1) = 6.215, *p* = 0.013), with laboratory studies having larger effects than non-laboratory studies (laboratory: *r* = 0.170, LLCI/ULCI = 0.125/0.215, *k* = 99; non-laboratory: *r* = 0.077, LLCI/ULCI = 0.033/0.121, *k* = 35).

*Single blind/double blind.* There was no moderating effect (QM (1) = 0.955, *p* = 0.328) of single or double blind on the observability–prosociality link (single blind: *r* = 0.130, LLCI/ULCI = 0.92/0.168, *k* = 106; double blind: *r* = 0.178, LLCI/ULCI = 0.087/0.268, *k* = 18).

*Nature of outcome.* There was no significant moderating effect of the nature of the outcome (QM (1) = 0.419, *p* = 0.517, whether to give: *r* = 0.131, LLCI/ULCI = 0.066/0.196, *k* = 29; how much to give: *r* = 0.161, LLCI/ULCI = 0.113/0.209, *k* = 78).

*Unearned versus earned.* Whether the endowment was earned or unearned was not a significant moderator (QM (1) = 1.374, *p* = 0.241) of the link between observability and prosociality (earned: *r* = 0.222, LLCI/ULCI = 0.065/0.379, *k* = 10; unearned: *r* = 0.133, LLCI/ULCI = 0.087/0.180, *k* = 91).

*Measures of outcome.* There was no significant moderating effect of subjective versus objective measures (QM (1) = 1.13, *p* = 0.288: subjective: *r* = 0.081, LLCI/ULCI = −0.010/0.172, *k* = 10; objective: *r* = 0.146, LLCI/ULCI = 0.109/0.183, *k* = 122).

*Type of prosocial behaviour.* The type of prosocial behaviour (monetary donations versus non-monetary donations) did not significantly moderate (QM (1) = 0.686, *p* = 0.408) the link between observability and prosocial behaviour (monetary: *r* = 0.151, LLCI/ULCI = 0.107/0.195, *k* = 101; non-monetary: *r* = 0.115, LLCI/ULCI = 0.067/0.163, *k* = 33).

*Aggregated data.* There was no significant moderating effect of aggregated versus non-aggregated data (QM (1) = 3.597, *p* = 0.058); however, studies that aggregated their data did have larger effects than non-aggregated data (non-aggregated: *r* = 0.133, LLCI/ULCI = 0.099/0.166, *k* = 129; aggregated: *r* = 0.290, LLCI/ULCI = 0.059/0.521, *k* = 5).

### (c) Meta-regression

A meta-regression was conducted on the significant moderators (see the electronic supplementary material, S4 Meta Regression and table S3). The effect of using single or repeated measures was the only significant predictor suggesting that those studies that used repeated measure generally reported larger effects (*r* = 0.14, LLCI/ULCI = 0.003/0.276.)

## 4. Discussion

The last three decades of research on the relationship between observability and prosociality have provided mixed findings, and we suggest this is owing to the inclusion or omission of key theoretical design features in the studies, that represent the key mechanisms facilitating the observability–prosociality link. These mark the boundary conditions for the observability–prosociality link and we report a meta-analytic review to examine these. In this review, we found observability had overall a small positive association with prosociality (*r* = 0.141). Four mechanisms of the decision-making context moderated this relationship. Three are consistent with predictions from CST, competitive altruism and reciprocity theory: (i) in contexts where participants make decisions with consequences for themselves (decisions that could influence how others respond to them), the effect of observability is larger, (ii) repeated games have larger effects on the observability–prosociality link than single games, and (iii) the effect of observability in social dilemmas was larger than in bargaining games. The fourth mechanistic finding, that the presence of passive observers strengthened the observability and prosociality link, is not consistent with theory. Finally, one methodological moderator was significant: the effect of observability was larger in laboratory studies than in field studies.

To contextualize the theoretical and practical interpretations of these findings, we should refer to the limitations of this meta-analysis. First, we applied mixed-effect models which can be overly conservative [58] owing to an overestimation of the sample error variance. This leads to confidence intervals that are too wide, and as a result, the amount of information gained from the meta-analysis is reduced [59]. However, adopting mixed-effect models makes sense because it assumes a degree of systematic variation (moderators) and some random population variation [58].

Second, the majority (80%, *k* = 94) of the studies in this meta-analysis come from western, industrialized countries; therefore, caution is needed when generalizing the effects of visibility on prosocial behaviour beyond western, educated, industrialized, rich and democratic societies [60].

Third, most of the studies (73%, *k* = 99) within the meta-analysis are experimental, with random allocation to condition and exogenous manipulations of observability which allows tentative inferences to be made regarding the causality of observability on enhancing prosocial behaviour.

### (a) Theoretical implications

The main contribution of the meta-analysis is to identify the boundary conditions for the observability–prosociality link. While we show that observability has a small positive and significant effect on prosociality, importantly we show that this varies significantly as a function of key mechanistic moderators suggested by theory (CST, competitive altruism and indirect reciprocity).

Moderator analyses showed that different types of observability manipulations (perceived, pseudo and overt) all have a small positive effect on prosocial behaviour, with slightly larger effects in pseudo and overt manipulations. Northover *et al*. [17] in their meta-analyses on the ‘watching eyes’ effect reported no overall effect of images of watching eyes on prosociality which is not comparable with the small effect of perceived visibility we report here. This is owing to our index of perceived visibility including studies that use manipulations other than watching eyes [61,62]. For example, Uziel & Hefetz [62] used a sentence completion task priming either a private or public mindset. In addition, Northover *et al*. [17] excluded some studies that are included in the current meta-analysis. For example, Northover *et al*. [17] excluded studies that reported their findings at aggregated levels (e.g. Powell *et al*. [63] report results per 1000s of customers). The effect of perceived manipulations could be susceptible to individual differences such as sensitivity to conformity and social pressure [64,65].

Three findings are consistent with predictions that observability enhances prosociality: (i) in contexts where participants make decisions with consequences for themselves, (ii) repeated games, and (iii) in social dilemmas.

The present findings indicate that decisions with consequences have a positive moderating influence on the observability–prosociality link compared to consequence-free decisions. In studies where decisions had consequences, participants' performance on one economic game could influence how others responded to them in a subsequent game [22,31]. Alternatively, participants’ previous contribution history were made observable to others [66]. This finding makes sense theoretically from cost signalling, competitive altruism or indirect reciprocity perspectives because, in circumstances where there are imminent future rewards, allocators should wish to positively advertise their cooperative qualities to increase the likelihood of being the beneficiary of those rewards [35,45]. Theoretically, the only danger of generosity in public context, where the observer knows that allocators are acting generously to secure future rewards, is that the signal could be perceived to be dishonest and could cast doubt over the cooperative qualities being signalled [22].

Repeated measure games had a stronger positive effect than single games. This can be explained from an indirect reciprocity perspective because repeated measure games allow individuals to build up reputations over time through consistent generous behaviour [67]. This benefits individuals with good reputations, because they are likely to receive more cooperative behaviour from others in the future [34]. Repeated interactions also build up a history of generous or selfish behaviour which can be used by others to more confidently judge whether this individual is someone whom they wish to interact with [34].

The results also showed that social dilemma games had a stronger effect on the observability–prosociality link compared with bargaining games. This effect could be attributable to social dilemmas providing greater opportunity to build reputations than bargaining games. For instance, social dilemma games, like the public goods game, are often played with multiple players (typically four players) while bargaining style games are often dyadic. Therefore, a typical public goods game under observable conditions allows players to signal their cooperation to three other players, while a typical dictator game gives the opportunity to signal their altruism to another player.

Not predicted by theory, we found that observer type was a substantial moderator of prosocial behaviour with passive observers (someone who is not participating in the experiment nor conducting the study) having a stronger effect on the observability–prosociality link than having no actual audience (the sense of being watched). While not predicted by theory, this moderating effect may be because of the high level of scrutiny allocators experienced during studies using passive observer designs [68]. For example, in Filiz-Ozbay & Ozbay [69]'s study, observers stood directly behind participants, watching them during a public goods game. Theoretically, this is interesting as it suggests that it is not just the question of whether a behaviour is observable, but also the intensity of observation that matters. Following the suggestion of one of the reviewers, an alternative explanation of the passive observer effect concerns the intensity and ambiguity of the observer's role. This may cause the participant to question what would happen if they met the observer outside of the laboratory context. Owing to the intensity of observation, the observer is likely to remember them beyond the experiment and might use their impression of them during the experiment to influence how they behave towards the participants in the real world.

There was one methodological moderator with laboratory studies having a stronger effect on the observability–prosociality link compared to non-laboratory studies. It is not unusual for there to be a disparity between behaviour within the laboratory or conducted within the field [40,70]. A possible explanation for this result is that manipulations of observability within the laboratory can be more tightly controlled, will be less subject to the influence of extraneous variables and hence be more effective than is possible within the field.

### (b) Practical implications

The main finding that observability enhances prosocial behaviour suggests that charities should continue to use observability manipulations as part of their fundraising strategies. In addition to charities, private organizations and public bodies should consider using observability manipulations as they have been found to affect a wide range of prosocial behaviour like increasing voter turnout, donating blood, volunteering and littering [10,45,71,72]. The moderator analyses suggest that certain features of observability manipulations are likely to produce larger effects. For example, framing the donation decision for the donor as a decision with consequences rather than as a consequence-free decision should enhance the impact of visibility on prosocial behaviour. This can be illustrated using the example of ‘The Ice-Bucket Challenge’. Individuals were nominated publically via social media to make a decision with a consequence: either they donated to the charity and performed the ice-bucket challenge (enduring water and ice thrown over themselves) or they refused and did not take part with the subsequent risk of losing face or reputation in front of peers on social networking sites [73].

## 5. Conclusion

Charities and researchers have shown a lot of interest in the effects of observability on prosocial behaviour. The main substantive finding of the current meta-analysis was the small positive effect that observability had on prosocial behaviour. Furthermore, this meta-analysis also revealed that studies that used particularly close observations of behaviour and gave allocators decisions with consequences tended to have the largest effects on prosociality.

## Supplementary Material

Does Observability Affect Prosociality: Supplementaries.

## Data accessibility

Coding framework, data and analysis script can be accessed at https://osf.io/87f4s/ [74].
