# Supplementary material for: Does observability affect prosociality?
Source: Proc Biol Sci. 2018 Mar 28;285(1875):20180116. doi: 10.1098/rspb.2018.0116 (PMC5897647; doi:10.1098/rspb.2018.0116)
Supplement: Does Observability Affect Prosociality: Supplementaries. [file rspb20180116supp1.docx]

Supplementary File S1 Overview of Studies

Table S1 details author, year, sample size, observability manipulation, type of prosocial behaviour and effect size of all the studies in the meta-analysis.

Table S1. The effect of Observability on Prosocial Behavior.

| Author (k =130) | Year | N | Type of Observability | Description of Observability Manipulation | Type of Prosocial Behaviour | r | 95% LL/ULCI |
| --- | --- | --- | --- | --- | --- | --- | --- |
| Alevy et al., | 2014 | 101 | Overt | Announcing donations in front of other dictators vs double-blind. | Monetary | 0.088 | 0.109/0.279 |
| Alpízar & Martinsson | 2013 | 1696 | Overt | Donations to the national park made alone or in the presence of a group or passive observer. | Monetary | 0.058 | 0.011/0.106 |
| Alpizar, Carlsson, & Johansson-Stenman | 2008 | 997 | Overt | Asking the participant to make a donation and bring it back to the solicitor to be recorded vs making a donation and putting the envelope in a sealed ballot box. | Monetary | 0.054 | -0.009/0.115 |
| Andreoni & Petrie | 2004 | 60 |  | Info (contribution but no identity information), photo (identity but no contribution information), photo and info (identity and contribution given)vs baseline condition (no identity or contribution), | Monetary | 0.140 | -0.118/0.381 |
| Ariely, Bracha, & Meier | 2009 | 77 | Overt | Informed that they will have to announce how much they donated to charity vs no announcement. | Monetary | -0.314 | -0.503/-0.097 |
| Baillon, Selim, & van Dolder | 2013 | 110 | Perceived | Eyes vs control Images | Monetary | 0.206 | 0.019/0.378 |
| Barclay | 2004 | 120 | Pseudo | Contributions to PGG written on a blackboard with pseudonyms and informed of the subsequent economic game vs contributions recorded but no information of the subsequent economic game. | Monetary | 0.209 | 0.031/0.374 |
| Barclay & Barker  Study 1. | 2015 | 36 | Overt | Donations are known (with and without partner choice) vs donation not known (control) | Monetary | 0.459 | 0.154/0.684 |
| Author (k =130) | **Year** | **N** | **Type of Observability** | **Description of Observability Manipulation** | **Type of Prosocial Behavior** | **r** | **95% LL/ULCI** |
| Barclay & Barker. Study 2. | 2015 | 36 | Overt | Donations are known (with and without partner choice) vs donation not known (control) | Monetary | 0.523 | 0.235/0.727 |
| Barclay & Willer | 2007 | 54 | Pseudo | Participants are informed that their donation decisions will be made public to passive observer vs kept private. | Monetary | 0.435 | 0.190/0.630 |
| Barmettler, Fehr, & Zehnder | 2012 | 201 | Pseudo | Single vs double anonymous pay-out systems. | Monetary | 0.043 | -0.096/0.180 |
| Bateson, Nettle, & Roberts | 2006 | 48 | Perceived | Eyes vs flower images above coffee honesty box | Monetary | 0.364 | 0.089/0.587 |
| Bateson et al., Study 1. | 2015 | 284 | Perceived | Eyes on a leaflet vs image of a lock on the leaflet. Leaflets placed over bike handlebars warning of the dangers of thieves. | Non-Monetary (Not littering) | 0.327 | 0.219/0.427 |
| Bateson et al., Study 2. | 2015 | 313 | Perceived | Eyes (large and small) on leaflet vs lock on leaflet placed over bike handlebars warning of the dangers of thieves. | Non-Monetary (Not littering) | 0.183 | 0.073/0.288 |
| Bereczkei, Birkas, & Kerekes | 2010 | 187 | Overt | Announcing volunteering intention to seminar group vs keeping volunteering intentions private. | Non-Monetary (Volunteering) | 0.235 | 0.095/0.366 |
| Bereczkei, Birkas, & Kerekes | 2010 | 194 | Overt | Announcing volunteering intention to seminar group vs keeping volunteering intentions private. | Non-Monetary (Volunteering) | 0.164 | 0.023/0.298 |
| Bereczkei, Birkas, & Kerekes | 2007 | 214 | Overt | Public offer of assistance in front of seminar group vs anonymous offers of assistance. | Non-Monetary (Volunteering) | 0.279 | 0.151/0.399 |
| Bihm, Gaudet, & Sale | 1979 | 205 | Overt | Lost letter technique. Observable if the car driver had passenger’s vs driving alone. | Non-Monetary (Effort) | -0.082 | -0.217/0.055 |
| Binzel & Fehr | 2013 | 36 | Overt | The identity of the dictators was revealed after the donation decision was made vs identity kept anonymously. | Monetary | 0.089 | -0.247/0.405 |
| Author (k =130) | **Year** | **N** | **Type of Observability** | **Description of Observability Manipulation** | **Type of Prosocial Behavior** | **r** | **95% LL/ULCI** |
| Böhm & Regner | 2013 | 58 | Overt | Rankings of participants earning task performance would be known by others vs not known. | Non-Monetary (Effort) | 0.336 | 0.085/0.547 |
| Bohnet & Frey | 1999a | 224 | Overt | Identity revealed to others (one way identification condition) and identity plus donation intentions (unrestricted communication condition) vs control condition (no identity revealed) | Monetary | 0.605 | 0.514/0.682 |
| Buhrmester, Goldfarb, & Cantrell | 1992 | 238 | Overt | Children informed their partner will find out how many tokens they gave vs double-blind condition. | Monetary | 0.151 | 0.024/0.273 |
| Burnham | 2003 | 60 | Overt | Dictators have their picture shown to the recipient vs no photo is shown in the control. | Monetary | 0.108 | -0.150/0.353 |
| Burnham & Hare | 2007 | 96 | Perceived | Picture of a robot with eyes vs no picture of a robot with eyes. | Monetary | 0.245 | 0.047/0.425 |
| Cadsby, Servátka, & Song | 2010 | 350 | Pseudo | Single vs double blind | Monetary | -0.028 | -0.133/0.077 |
| Carbon & Hesslinger | 2011 | 138 | Perceived | Eyes vs flower images. | Non-Monetary (Prosocial Intentions) | 0.050 | -0.118/0.215 |
| Carpenter & Myers | 2010 | 320 | Pseudo | Car numberplate proxy for image vs no car number plate. | Non-Monetary (Volunteering) | 0.095 | -0.015/0.203 |
| Casal & Mittone | 2014 | 144 | Overt | Subject photos were displayed vs anonymity in the baseline and control conditions. | Monetary | 0.145 | -0.019/0.302 |
| Chetty, Saez, & Sándor | 2014 | 779 | Overt | Names and length of time taken to review papers published on a website | Non-Monetary (Time reviewing Manuscripts) | -0.026 | -0.096/0.045 |
| Dufwenberg & Muren | 2006 | 310 | Overt | Dictator told they will either collect money on stage in front of other 1st-year economics students or in private. | Monetary | -0.216 | -0.320/-0.107 |
| Author (k =130) | **Year** | **N** | **Type of Observability** | **Description of Observability Manipulation** | **Type of Prosocial Behavior** | **r** | **95% LL/ULCI** |
| Ekström | 2012 | 408 | Perceived | Eyes vs control image over supermarket recycling machines. | Monetary | -0.003 | -0.100/0.095 |
| Engelmann, Herrmann & Tomasello | 2012 | 48 | Overt | Children were watched by another unknown 5-year-old vs not watched in the private condition | Monetary | 0.216 | -0.072/0.471 |
| Ernest-Jones, Nettle, & Bateson | 2011 | 562 | Perceived | Eyes vs control Image | Non-Monetary (Not Littering) | 0.163 | 0.082/0.243 |
| Everett, Faber, & Crockett. Study 1. | 2015 | 29 | Pseudo | In group identity and pay off shown. | Monetary | 0.211 | -0.169/0.540 |
| Everett, Faber, & Crockett. Study 2. | 2015 | 30 | Pseudo | In group identity and pay off shown. | Monetary | 0.176 | -0.197/0.504 |
| Fathi, Bateson, & Nettle | 2014 | 123 | Perceived | Picture of eyes vs no image of eyes. | Monetary | 0.156 | -0.022/0.324 |
| Fehr & Schneider | 2010 | 99 |  | Eyes and trustee aware of trustors previous transfers. | Monetary | 0.087 | -0.113/0.279 |
| Festre & Garrouste | 2014 | 132 | Pseudo | The observer is informed about how much the dictator gave. | Monetary | 0.110 | -0.062/0.276 |
| Filiz-Ozbay & Ozbay | 2014 | 288 | Overt | Observers watch their performance. | Non-Monetary (Effort Task) | 0.471 | 0.376/0.556 |
| Fox & Guyer | 1978 | 80 | Overt | Identity (see each other through mirrors) and donation amounts visible (at the end of the round) vs no identity and donation information. | Monetary | 0.312 | 0.099/0.498 |
| Francey & Bergmuller | 2012 | 60 | Perceived | Eyes vs control Images. | Non-Monetary (Tidying up rubbish) | 0.272 | 0.019/0.492 |
| Franzen & Pointner | 2012 | 177 | Perceived | Randomized Response Technique vs double blind in DG. | Monetary | 0.502 | 0.382/0.604 |
| Gabriel, Banse, & Hug | 2007 | 69 | Overt | Experimenter in the room with them vs experimenter not in the room. | Monetary | 0.474 | 0.268/0.639 |
| Gächter & Fehr | 1999 | 116 | Overt | Participants had identity and contribution revealed and had to discuss their decisions with other group members vs never learn each other’s identity (control). | Monetary | 0.036 | -0.148/0.217 |
| Author (k =130) | **Year** | **N** | **Type of Observability** | **Description of Observability Manipulation** | **Type of Prosocial Behavior** | **r** | **95% LL/ULCI** |
| Gerber, Green, & Larimer | 2008 | 229451 | Overt | Hawthorne group (informed researchers are watching), self-group (informed voting records are public and their household would be mailed with information of who voted), neighbours (list households and nearby households voting records with threat that vote record will be distributed to self and neighbour) vs control group (received no message) | Non-Monetary (Voting) | 0.059 | 0.055/0.063 |
| Gerber, Green, & Larimer | 2010 | 359657 | Overt | Past voting (showed participant all those in household who had voted), past absentation (showed all those in household who had not voted) vs control group (received no message), | Non-Monetary (Voting) | 0.023 | 0.020/0.026 |
| Grimalda, Pondorfer, & Tracer | 2016 | 114 | Overt | Big man (person of authority) was watching their allocations in the prisoner's dilemma vs no one watching | Monetary | 0.067 | -0.118/0.248 |
| Haley & Fessler | 2005 | 124 | Perceived | Stylized eyes on the desktop of computer vs control image on the desktop of the computer. | Monetary | 0.239 | 0.066/0.399 |
| Hardy & Van Vugt Study 1. | 2006 | 66 | Pseudo | Feedback sheets detailed individual’s contributions in the previous round vs no feedback sheets. | Monetary | 0.193 | -0.052/0.416 |
| Hardy & Van Vugt Study 2. | 2006 | 150 | Pseudo | Feedback sheets detailed individual’s contributions in the previous round vs no feedback sheets. | Monetary | 0.210 | 0.051/0.358 |
| Hauser, Hendriks, Rand, & Nowak | 2016 | 646 | Pseudo | See ID and contribution to PGG. | Monetary | 0.336 | 0.267/0.403 |
| Hoffman, Mccabe, & Smith | 1996 | 157 | Pseudo | Single vs Double-blind | Monetary | 0.068 | -0.089/0.223 |
| Hoffman, McCabe, Shachat, & Smith | 1994 | 60 | Pseudo | Single vs Double-blind | Monetary | 0.458 | 0.230/0.637 |
| Horita & Takezawa | 2014 | 44 |  | Experimenter watching their decision vs participant remaining anonymous from the experimenter. | Monetary | 0.026 | -0.273/0.321 |
| Author (k =130) | **Year** | **N** | **Type of Observability** | **Description of Observability Manipulation** | **Type of Prosocial Behavior** | **r** | **95% LL/ULCI** |
| Huang, Liu, & Liu | 2016 | 88 | Overt | Decision and amount they donated released on the website vs private and confidential decision. | Non-Monetary (Intentions) | -0.124 | -0.325/0.088 |
| Hubbard et al. | 2016 | 80 | Overt | A passive observer would be observing their choices on public trials vs no observers in private trials. | Monetary | 0.522 | 0.341/0.665 |
| Hugh-Jones & Reinstein | 2009 | 130 | Pseudo | Donations made by players either revealed with player ID vs total number of contributions to the PG revealed (control) | Monetary | -0.124 | -0.290/0.049 |
| Iredale, Vugt, & Dunbar Study 1. | 2008 | 30 | Overt | Observer of the same or opposite sex vs no observer | Monetary | 0.136 | -0.236/0.473 |
| Iredale, Vugt, & Dunbar Study 2. | 2008 | 72 | Pseudo | Decisions made public to other players vs decisions kept anonymous | Monetary | 0.310 | 0.085/0.506 |
| Izuma, Saito, & Sadato | 2010 | 23 | Overt | Video of two observers watching them | Monetary | 0.532 | 0.153/0.774 |
| Izuma, Matsumoto, Camerer, & Adolphs | 2011 | 11 | Overt | A passive observer would be observing their choices on public trials vs no observers in private trials. | Monetary | 0.685 | 0.145/0.911 |
| Jacquet, Hauert, Traulsen, & Milinski | 2011 | 60 | Overt | Top (honour) and bottom (shame) contributors had name published at the end of the study. | Monetary | 0.571 | 0.372/0.403 |
| Jerdee & Rosen | 1974 | 180 | Pseudo | The amount of profit earned by a participant is displayed on a blackboard vs kept private (control). | Monetary | 0.284 | 0.144/0.413 |
| Jolij & de Haan | 2014 | 103 | Perceived | Images of Eyes vs Flower images | Monetary | 0.068 | -0.127/0.258 |
| Jones & Linardi | 2014 | 150 | Overt | A public ceremony of each participants donations to other participants vs no ceremony. | Monetary | 0.115 | -0.047/0.270 |
| Karlan & McConnell Study 2. | 2014 | 94 | Overt | Name and amount donated by each participant wrote on the blackboard vs private 1^st^ round decisions. | Monetary | 0.252 | 0.052/0.432 |
| Kataria & Regner | 2015 | 185 | Overt | Public ceremony of effort task performance (ranking and scores given) | Non-Monetary (Effort Task) | 0.132 | -0.013/0.271 |
| Matsugasaki, Tsukamoto, & Ohtsuo Study 1. | 2015 | 33 | Perceived | Eyes on Posters (male or female) or Geometric patterns. | Monetary | -0.166 | -0.482/0.188 |
| Author (k =130) | **Year** | **N** | **Type of Observability** | **Description of Observability Manipulation** | **Type of Prosocial Behavior** | **r** | **95% LL/ULCI** |
| Matsugasaki, Tsukamoto, & Ohtsubo Study 2. | 2015 | 49 | Perceived | Eyes on envelopes participants placed money in or geometric patterns on envelopes. | Monetary | -0.103 | -0.374/0.183 |
| Keller & Pfattheicher | 2011 | 50 | Perceived | Watching eyes or blank headed paper. | Monetary | -0.415 | -0.621/-0.154 |
| Kratky, McGraw, Xygalatas, Mitkidis, & Reddish | 2016 | 270 | Perceived | 3d and 2d image of eyes vs control 2d/3d image of the plant | Monetary | 0.087 | -0.032/0.204 |
| Kraus & Callaghan Study 2. | 2016 | 676 | Overt | Participants donation in DG were accompanied by their name and city they lived in (public condition) | Monetary | -0.051 | -0.125/0.025 |
| Kraus & Callaghan Study 3. | 2016 | 361 | Overt | Participants donation in DG were accompanied by their name and city they lived in (public condition) | Monetary | 0.080 | -0.024/0.182 |
| Kurzban | 2001 | 289 |  | Identity information revealed vs no identity information revealed. | Monetary | 0.237 | 0.126/0.343 |
| Lacetera & Macis | 2010 | 914 | Overt | Public ceremony and name published in newsletter those who donate 50th,75th and 100th time. | Non-Monetary (Donate Blood) | 0.145 | 0.081/0.208 |
| Lamba & Mace | 2010 | 55 | Overt | Identity and donation revealed to other participants at the end. | Monetary | 0.213 | -0.056/0.452 |
| Lambarraa & Riener | 2015 | 357 | Overt | Publicly announced name and size of donation | Monetary | -0.105 | -0.207/-0.001 |
| Laury, Walker, & Williams | 1995 | 35 | Pseudo | Single vs Double blind | Monetary | 0.324 | -0.011/0.593 |
| Leimgruber, Shaw, Santos, & Olson | 2012 | 32 | Overt | Children either visible to partner or occluded from view of partner whilst making decisions | Monetary | 0.554 | 0.255/0.757 |
| Li & Riyanto | 2017 | 244 | Overt | High contributing donors were asked to stand up in front of the group and had a star placed on the seating plan so other knew where they were vs anonymous donations. | Monetary | 0.066 | -0.061/0.190 |
| Manesi, Van Lange, & Pollet Study 1. | 2016 | 148 | Perceived | Eyes gazing straight at you, vs flowers image. | Non-Monetary (Effort task) | 0.189 | 0.028/0.340 |
| Author (k =130) | **Year** | **N** | **Type of Observability** | **Description of Observability Manipulation** | **Type of Prosocial Behavior** | **r** | **95% LL/ULCI** |
| Manesi, Van Lange, & Pollet Study 2. | 2016 | 113 | Perceived | Eyes gazing straight at you, vs flowers image. | Non-Monetary (Effort task) | 0.152 | -0.034/0.327 |
| Manesi, Van Lange, & Pollet Study 3. | 2016 | 173 | Perceived | Eyes gazing straight at you, vs flowers image. | Non-Monetary (Effort task) | 0.082 | -0.068/0.228 |
| Martinsson & Villegas-palacio | 2010 | 96 | Overt | Three observability treatments that have been combined (revealing donation to an in-group, an outgroup or to both in-group and out-group). In each case participants stood up announced their ID and their donation amount. In the control both identity and amount donated are anonymous. | Monetary | 0.102 | -0.100/0.297 |
| Mason | 2016 | 4486 | Overt | Names published in the newsletter vs names not published | Non-Monetary (intent to participate in fundraising campaign) | 0.081 | 0.052/0.110 |
| Matland & Murray | 2015 | 60239 | Perceived | Eyes on postcard reminding them to vote vs no postcard (control). | Non-Monetary (Voting) | 0.006 | -0.002/0.014 |
| Mifune, Hashimoto, & Yamagishi | 2010 | 140 | Perceived | Stylized eyes vs green background on the computer. | Monetary | 0.009 | -0.158/0.174 |
| Nettle et al. | 2013 | 118 | Perceived | Poster of eyes or University Logo | Monetary | 0.100 | -0.082/0.276 |
| Noussair & Tucker | 2007 | 24 | Overt | Participant identity and contributions revealed vs kept private. | Monetary | -0.310 | -0.634/0.107 |
| Oda & Ichihashi | 2016 | 8189 | Perceived | Eyes on perspex box in Japanese tavern vs control image (butterfly) | Monetary | 0.036 | 0.015/0.058 |
| Oda, Niwa, Honma, & Hiraishi | 2011 | 62 | Perceived | Eyes vs no Image. | Monetary | 0.284 | 0.037/0.498 |
| Panagopoulos | 2014 | 24531 | Perceived | Voters showed postcard with eyes on vs control images. | Non-Monetary (Voting) | 0.028 | 0.015/0.040 |
| Panagopoulos | 2014 | 5514 | Perceived | Voters shown postcard with eyes on vs receive no postcard or postcard with control images. | Non-Monetary (Voting) | 0.081 | 0.055/0.107 |
| Author (k =130) | **Year** | **N** | **Type of Observability** | **Description of Observability Manipulation** | **Type of Prosocial Behavior** | **r** | **95% LL/ULCI** |
| Panagopoulos | 2010 | 19400 | Overt | Postcards informing them their names may be published in local newspaper vs no postcard sent (control) | Non-Monetary (Voting) | 0.020 | 0.006/0.034 |
| Pedersen | 2016 | 189 | Perceived | Eyes vs message to complete the survey. | Non-Monetary (Completing Survey) | 0.053 | -0.091/0.194 |
| Pfattheicher & Keller | 2015 | 126 | Perceived | Stylized Eyes printed on top of a sheet of paper vs no subtle cue of being watched. | Monetary | 0.058 | -0.118/0.231 |
| Pfattheicher Study 2 Sample 1. | 2015 | 100 | Perceived | Picture of eyes vs no image of eyes. | Monetary | -0.064 | -0.257/0.134 |
| Pfattheicher Study 2 Sample 2. | 2015 | 123 | Perceived | Picture of eyes vs no image of eyes. | Monetary | 0.116 | -0.062/0.287 |
| Piazza & Bering | 2008 | 36 | Overt | Receiver will inform the third party of their behaviour | Monetary | 0.154 | -0.184/0.459 |
| Powell, Roberts, & Nettle | 2012 | 51893 | Perceived | Eyes vs control images of stars placed on charity buckets located at the end supermarket tills. | Monetary | 0.451 | 0.444/0.458 |
| Raihani & Bshary | 2012 | 191 | Perceived | Eyes vs control Image | Monetary | -0.141 | -0.277/0.001 |
| Reinstein & Riener | 2012 | 122 |  | Identity and donation revealed to other dictators vs No information revealed. | Monetary | 0.137 | -0.041/0.308 |
| Rigdon, Ishii, Watabe, & Kitayama | 2009 | 113 | Perceived | Three dots configured like eyes vs three dots in a neutral configuration. | Monetary | 0.174 | -0.011/0.348 |
| Roth | 1995 | 287 | Overt | Identity revealed (restricted communication) or identity and information revealed (unrestricted communication) vs no identity and donation information revealed (control condition). | Monetary | 0.014 | -0.102/0.130 |
| Samek, Sheremeta, & Angeles | 2015 | 205 | Overt | Names and donation amounts vs no information in control. | Monetary | 0.157 | 0.020/0.288 |
| Samek & Sheremeta | 2016 | 80 | Overt | First name and photo displayed with the amount donated vs ID and amount in the control group. | Monetary | 0.524 | 0.343/0.667 |
| Samek & Sheremeta | 2014 | 80 | Overt | Name and picture with amount and rank vs no identity information and amount donation (control) | Monetary | 0.561 | 0.389/0.695 |
| Author (k =130) | **Year** | **N** | **Type of Observability** | **Description of Observability Manipulation** | **Type of Prosocial Behavior** | **r** | **95% LL/ULCI** |
| Satow | 1975 | 80 | Overt | One way mirror with the experimenter behind the mirror and the participant table facing the mirror vs no one-way mirror and the table faced the door (control) | Monetary | 0.294 | 0.079/0.482 |
| Saunders, Taylor, & Atkinson | 2016 | 1535 | Perceived | Visible (eyes, faces, emotional faces) vs Control images(schematic inverted faces and black/white control squares) | Monetary | 0.004 | -0.046/0.054 |
| Schmidt & Zultan | 2005 | 48 | Overt | No Communication (no communication with the recipient and no information about them), restricted communication (communication with recipient but unaware of the game they are about to play) and unrestricted communication (game specific instruction provided before the interaction with the recipient and before the game). | Monetary | 0.079 | -0.210/0.355 |
| Sénémeaud et al., | 2017 | 454 | Perceived | Picture of eyes on blood donation leaflet vs control image | Non-Monetary (blood donating) | 0.179 | 0.088/0.266 |
| Smith et al., | 2009 | 78 | Overt | Informed they are visible to a second player and can also see the second player vs not visible to the second player. | Monetary | 0.315 | 0.099/0.502 |
| Sparks & Barclay | 2014 | 124 | Perceived | Eyes vs no Eyes | Monetary | 0.034 | -0.143/0.209 |
| Sparks Study 1. | 2010 | 106 | Perceived | Eyes vs landscapes images. | Monetary | -0.102 | -0.287/0.091 |
| Sparks Study 2. | 2010 | 83 | Perceived | Eyes vs landscapes images. | Monetary | 0.020 | -0.196/0.235 |
| Sparks Study 3. | 2010 | 107 | Perceived | Eyes vs landscapes images. | Monetary | -0.237 | -0.408/-0.049 |
| Tane & Takezawa | 2011 | 40 | Perceived | Eyes vs butterfly images | Monetary | -0.218 | -0.496/0.100 |
| Tane, & Takezawa | 2011 | 40 | Perceived | Eyes image vs blank screen | Monetary | -0.064 | -0.3680.252 |
| Thielmann, Heck, & Hilbig | 2016 | 950 | Pseudo | Single blind (public) vs Randomised response technique (control) | Monetary | 0.148 | 0.085/0.209 |
| Uziel & Hefetz. Study 1. | 2014 | 72 | Perceived | Priming private and public mindset through sentence completion | Monetary | -0.144 | -0.363/0.091 |
| Author (k =130) | **Year** | **N** | **Type of Observability** | **Description of Observability Manipulation** | **Type of Prosocial Behavior** | **r** | **95% LL/ULCI** |
| Uziel, & Hefetz. Study 2. | 2014 | 78 | Perceived | Priming private and public mindset through sentence completion | Monetary | -0.011 | -0.233/0.212 |
| van Bommel, van Prooijen, Elffers, & van Lange | 2014 | 80 | Overt | Participant has a camera either watching them or bystander vs no camera or no bystander. | Non-Monetary (preventing crime) | -0.362 | -0.539/-0.155 |
| van Rompay, Vonk, & Fransen | 2009 | 80 | Overt | Security camera watching them vs no camera. | Non-Monetary (Effort Task) | 0.332 | 0.121/0.514 |
| van Vugt & Hardy 1. | 2010 | 86 | Pseudo | Decisions made public to other players vs decisions kept anonymous | Monetary | 0.357 | 0.157/0.529 |
| Van Vugt & Iredale Study 1 | 2013 | 130 | Overt | Observer watching contributions vs no observers watching. | Monetary | 0.180 | 0.008/0.342 |
| van Vugt, & Iredale. Study 2. | 2013 | 47 | Overt | Observer watching contributions vs no observers watching. | Monetary and Non Monetary (intention to volunteer) | 0.176 | -0.117/0.441 |
| Vogt, Efferson, Berger, & Fehr | 2015 | 177 | Perceived | Children watched by stylized eyes vs images of perpendicular lines. | Monetary | -0.035 | -0.182/0.113 |
| Wichman | 1970 | 88 | Overt | Identity revealed (see condition), and identity and donation revealed (unrestricted communication) vs control condition (no identity or donation information), | Monetary | 0.248 | 0.040/0.435 |
| Vrouwe | 2014 | 95 | Perceived | Male or female images of eyes vs abstract art images. | Non-Monetary (Number of essay corrected) | 0.271 | 0.073/0.448 |
| Wilson & Sell | 1997 | 108 | Pseudo | Announcement (players anonymous but ID is displayed with intention to donate to public pot), History (ID displayed with past contributions), Control (no announcements and no history displayed) | Monetary | -0.330 | -0.489/-0.151 |
| Xin, Liu, Yang, & Zhang | 2016 | 235 | Perceived | Image of three dots configured either like eyes or reversed like triangles. | Monetary | 0.190 | 0.063/0.310 |
| Author (k =130) | **Year** | **N** | **Type of Observability** | **Description of Observability Manipulation** | **Type of Prosocial Behavior** | **r** | **95% LL/ULCI** |
| Xin, Liu, Yang, & Zhang | 2016 | 50 | Perceived | Image of three dots configured either like eyes or reversed like triangles. | Monetary | 0.383 | 0.117/0.598 |
| Xin, Liu, Yang, & Zhang | 2016 | 60 | Perceived | Image of three dots configured either like eyes or reversed like triangles. | Monetary | 0.289 | 0.038/0.506 |
| Yoeli, Hoffman, Rand, & Nowak | 2013 | 1408 | Overt | Sign up sheets either contained their name and residential address (visible) or anonymous code (Control) | Non-Monetary (participation in demand response system) | 0.069 | 0.016/0.120 |

References

1. Alevy JE, Jeffries FL, Lu Y (2014) Gender- and frame-specific audience effects in dictator games. *Econ Lett* 122(1):50–54.

2. Alpízar F, Martinsson P (2013) Does It Matter if You Are Observed by Others? Evidence from Donations in the Field. *Scand J Econ* 115(1):74–83.

3. Alpizar F, Carlsson F, Johansson-Stenman O (2008) Anonymity, reciprocity, and conformity: Evidence from voluntary contributions to a national park in Costa Rica. *J Public Econ* 92(5–6):1047–1060.

4. Andreoni J, Petrie R (2004) Public goods experiments without confidentiality: a glimpse into fund-raising. *J Public Econ* 88(7–8):1605–1623.

5. Ariely D, Bracha A, Meier S (2009) Doing Good or Doing Well?: Imge Motivation adn Monetary Incentives in Behaving Proscially. *Am Econ Rev* 99:544–555.

6. Baillon A, Selim A, van Dolder D (2013) On the social nature of eyes: The effect of social cues in interaction and individual choice tasks. *Evol Hum Behav* 34(2):146–154.

7. Barclay P (2004) Trustworthiness and competitive altruism can also solve the “tragedy of the commons.” *Evol Hum Behav* 25(4):209–220.

8. Barclay P, Barker J (2015) Greener than thou: partner choice creates competition to save the environment. *Human Behavior & Evolution Society (HBES)*.

9. Barclay P, Willer R (2007) Partner choice creates competitive altruism in humans. *Proc Biol Sci* 274(1610):749–53.

10. Barmettler F, Fehr E, Zehnder C (2012) Big experimenter is watching you! Anonymity and prosocial behavior in the laboratory. *Games Econ Behav* 75(1):17–34.

11. Bateson M, Nettle D, Roberts G (2006) Cues of being watched enhance cooperation in a real-world setting. *Biol Lett* 2(3):412–4.

12. Bateson M, et al. (2015) Watching eyes on potential litter can reduce littering: evidence from two field experiments. *PeerJ* 3:e1443.

13. Bereczkei T, Birkas B, Kerekes Z (2010) Altruism towards strangers in need: costly signaling in an industrial society. *Evol Hum Behav* 31(2):95–103.

14. Bereczkei T, Birkas B, Kerekes Z (2010) The presence of others, prosocial traits, Machiavellianism : A personality × situation approach. *Soc Psychol (Gott)* 41(4):238–245.

15. Bereczkei T, Birkas B, Kerekes Z (2007) Public charity offer as a proximate factor of evolved reputation-building strategy: an experimental analysis of a real-life situation. *Evol Hum Behav* 28(4):277–284.

16. Bihm E, Gaudet I, Sale O (1979) Altrusitic Response under conditions of anonymity. *J Soc Psychol* 109:25–30.

17. Binzel C, Fehr D (2013) Giving and sorting among friends: Evidence from a lab-in-the-field experiment. *Econ Lett* 121(2):214–217.

18. Böhm R, Regner T (2013) Charitable giving among females and males: an empirical test of the competitive altruism hypothesis. *J Bioeconomics* 15(3):251–267.

19. Bohnet I, Frey BS (1999) The sound of silence in prisoner’s dilemma and dictator games. *J Econ Behav Organ* 38(1):43–57.

20. Buhrmester D, Goldfarb J, Cantrell D (1992) Self-Presentation when sharing with friends and nonfriends. *J Early Adolesc* 12(1):61–79.

21. Burnham TC (2003) Engineering altruism: a theoretical and experimental investigation of anonymity and gift giving. *J Econ Behav Organ* 50(1):133–144.

22. Burnham TC, Hare B (2007) Engineering human cooperation : Does involuntary neural activation increase public goods contributions? *Hum Nat* 18(2):88–108.

23. Cadsby CB, Servátka M, Song F (2010) Gender and generosity: Does degree of anonymity or group gender composition matter? *Exp Econ* 13(3):299–308.

24. Carbon CC, Hesslinger VM (2011) Bateson et al.’s (2006) Cues-of-being-watched paradigm revisited. *Swiss J Psychol* 70(4):203–210.

25. Carpenter J, Myers CK (2010) Why volunteer? Evidence on the role of altruism, image, and incentives. *J Public Econ* 94(11–12):911–920.

26. Casal S, Mittone L (2014) Social esteem versus social stigma: The role of anonymity in an income reporting game. *J Econ Behav Organ* 124:55–66.

27. Chetty R, Saez E, Sándor L (2014) What Policies Increase Prosocial Behavior? An Experiment with Referees at the Journal of Public Economics. *J Econ Perspect* 28(3):169–188.

28. Dufwenberg M, Muren A (2006) Generosity, anonymity, gender. *J Econ Behav Organ* 61(1):42–49.

29. Ekström M (2012) Do watching eyes affect charitable giving? Evidence from a field experiment. *Exp Econ* 15(3):530–546.

30. Engelmann JM, Herrmann E, Tomasello M (2012) Five-Year Olds, but Not Chimpanzees, Attempt to Manage Their Reputations. *PLoS One* 7(10). doi:10.1371/journal.pone.0048433.

31. Ernest-Jones M, Nettle D, Bateson M (2011) Effects of eye images on everyday cooperative behavior: A field experiment. *Evol Hum Behav* 32(3):172–178.

32. Everett JAC, Faber NS, Crockett MJ (2015) The influence of social preferences and reputational concerns on intergroup prosocial behaviour in gains and losses contexts. *R Soc Open Sci* 2(12):150546.

33. Fathi M, Bateson M, Nettle D (2014) Effects of watching eyes and norm cues on charitable giving in a surreptitious behavioral experiment. *Evol Psychol* 12(5):878–887.

34. Fehr E, Schneider F (2010) Eyes are on us, but nobody cares: are eye cues relevant for strong reciprocity? *Proc Biol Sci* 277(1686):1315–1323.

35. Festre A, Garrouste P (2014) Somebody may scold you! A dictator experiment. *J Econ Psychol* 45:141–153.

36. Filiz-Ozbay E, Ozbay EY (2014) Effect of an audience in public goods provision. *Exp Econ* 17(2):200–214.

37. Fox J, Guyer M (1978) “ Public ” Choice and Cooperation in n-Person Prisoner â€^TM^ s Dilemma. *J Confl Resolut* 22(3):469–481.

38. Francey D, Bergmuller R (2012) Images of Eyes Enhance Investments in a Real-Life Public Good. *PLoS One* 7(5):1–7.

39. Franzen A, Pointner S (2012) Anonymity in the dictator game revisited. *J Econ Behav Organ* 81(1):74–81.

40. Gabriel U, Banse R, Hug F (2007) Predicting private and public helping behaviour by implicit attitudes and the motivation to control prejudiced reactions. *Br J Soc Psychol* 46(2):365–382.

41. Gächter S, Fehr E (1999) Collective action as a social exchange. *J Econ Behav Organ* 39(4):341–369.

42. Gerber A, Green D, Larimer C (2008) Social Pressure and Voter Turnout: Evidence from a Large-Scale Field Experiment. *Am Polit Sci Rev* 102(1):16.

43. Gerber A, Green D, Larimer C (2010) An Experiment Testing the Relative Effectiveness of Encouraging Voter Participation by Inducing Feelings of Pride or Shame . *Polit Behav* 32(3):409–422.

44. Grimalda G, Pondorfer A, Tracer DP (2016) Social image concerns promote cooperation more than altruistic punishment. *Nat Commun* 7:1–8.

45. Haley KJ, Fessler DMT (2005) Nobody’s watching? Subtle cues affect generosity in an anonymous economic game. *Evol Hum Behav* 26(3):245–256.

46. Hardy CL, Van Vugt M (2006) Nice Guys Finish First: The Competitive Altruism Hypothesis. *Personal Soc Psychol Bull* 32(10):1402–1413.

47. Hauser OP, Hendriks A, Rand DG, Nowak MA (2016) Think global, act local: Preserving the global commons. *Sci Rep* 6(1):36079.

48. Hoffman BE, Mccabe K, Smith VL (1996) Social Distance and Other-Regarding Behavior in Dictator Games Author ( s ): Elizabeth Hoffman , Kevin McCabe and Vernon L . Smith Source : The American Economic Review , Vol . 86 , No . 3 ( Jun ., 1996 ), pp . 653-660 Publis. *Am Econ Assoc* 86(3):653–660.

49. Hoffman E, McCabe K, Shachat K, Smith V (1994) Preferences, Property Rights, and Anonymity in Bargaining Games. *Games Econ Behav* 7(3):346–380.

50. Horita Y, Takezawa M (2014) Observation Enhances Third-Party Punishment Only among People Who Were not Hot-Tempered. *Lett Evol Behav Sci* 5(1):5–8.

51. Huang H, Liu Y, Liu X (2016) Does loneliness necessarily lead to a decrease in prosocial behavior? The roles of gender and situation. *Front Psychol* 7(SEP):1–9.

52. Hubbard J, et al. (2016) A General Benevolence Dimension That Links Neural , Psychological , Economic , and Life-Span Data on Altruistic Tendencies. doi:10.1037/xge0000209.

53. Hugh-Jones D, Reinstein D (2009) Secret Santa: Anonymity, Signaling, and Conditional Cooperation. *Jena Econ Res Pap* 2009:1–55.

54. Iredale W, Vugt M Van, Dunbar R (2008) Showing off in humans: Male generosity as a mating signal. *Evol Psychol* 6(3):386–392.

55. Izuma K, Saito DN, Sadato N (2010) Processing of the incentive for social approval in the ventral striatum during charitable donation. *J Cogn Neurosci* 22(4):621–631.

56. Izuma K, Matsumoto K, Camerer CF, Adolphs R (2011) Insensitivity to social reputation in autism. *Proc Natl Acad Sci U S A* 108(42):17302–7.

57. Jacquet J, Hauert C, Traulsen a., Milinski M (2011) Shame and honour drive cooperation. *Biol Lett* 7(6):899–901.

58. Jerdee TH, Rosen B (1974) Effects of opportunity to communicate and visibility of individual decisions on behavior in the common interest. *J Appl Psychol* 59(6):712–716.

59. Jolij J, de Haan T (2014) *Being watched doesn’t make you nicer: no effect of visible and invisible eye primes on prosocial behavior in a masked priming study*.

60. Jones D, Linardi S (2014) Wallflowers: Experimental Evidence of an Aversion to Standing Out. *Manage Sci* (online):1–16.

61. Karlan D, McConnell M (2014) Hey look at me: The effect of giving circles on giving. *J Econ Behav Organ* 106:402–412.

62. Kataria M, Regner T (2014) Honestly, why are you donating money to charity? An experimental study about self-awareness in status-seeking behavior. *Theory Decis* 79(3):493–515.

63. Matsugasaki K, Tsukamoto W, Ohtsubo Y (2015) *Two failed replications of the watching eyes effect.*

64. Keller J, Pfattheicher S (2011) Vigilant self regulation, cues of being watched and cooperativeneess. *Eur J Pers* 25:363–372.

65. Kratky J, McGraw JJ, Xygalatas D, Mitkidis P, Reddish P (2016) It depends who is watching you: 3-D agent cues increase fairness. *PLoS One* 11(2):1–11.

66. Kraus MW, Callaghan B (2016) Social class and prosocial behavior: The moderating role of public versus private contexts. *Soc Psychol Personal Sci* 7(8):1948550616659120.

67. Kurzban R (2001) The Social Psychophysics of Cooperation: Nonverbal Communication in a public good game. *J Nonverbal Behav* 25(4):241–259.

68. Lacetera N, Macis M (2010) Social image concerns and prosocial behavior: Field evidence from a nonlinear incentive scheme. *J Econ Behav Organ* 76(2):225–237.

69. Lamba S, Mace R (2010) People recognise when they are really anonymous in an economic game. *Evol Hum Behav* 31(4):271–278.

70. Lambarraa F, Riener G (2015) On the norms of charitable giving in Islam: Two field experiments in Morocco. *J Econ Behav Organ* 118:69–84.

71. Laury SK, Walker JM, Williams AW (1995) Anonymity and the voluntary provision of public goods. *J Econ Behav Organ* 27(3):365–380.

72. Leimgruber KL, Shaw A, Santos LR, Olson KR (2012) Young Children Are More Generous when Others Are Aware of Their Actions. *PLoS One* 7(10). doi:10.1371/journal.pone.0048292.

73. Li J, Riyanto YE (2017) Category Reporting in Charitable Giving: an Experimental Analysis. *Econ Inq* 55(1):397–408.

74. Manesi Z, Van Lange PAM, Pollet TV (2016) Eyes Wide Open: Only Eyes That Pay Attention Promote Prosociality. *Evol Psychol* (Advance online publication):1–15.

75. Martinsson P, Villegas-palacio C (2010) *Does disclosure crowd out cooperation ?* Available at: file:///D:/Archivos Dropbox/Dropbox/Papers/Martinsson, Villegas-palacio/Working Papers in economics No.446/Martinsson, Villegas-palacio - 2010 - Does disclosure crowd out cooperation Does disclosure crowd out cooperation.pdf.

76. Mason DP (2016) Recognition and Cross-Cultural Communications as Motivators for Charitable Giving A Field Experiment. *Nonprofit Volunt Sect Q* 45(1):192–2014.

77. Matland RE, Murray GR (2015) I Only Have Eyes for You: Does Implicit Social Pressure Increase Voter Turnout? *Polit Psychol* 37(4):533–550.

78. Mifune N, Hashimoto H, Yamagishi T (2010) Altruism toward in-group members as a reputation mechanism. *Evol Hum Behav* 31(2):109–117.

79. Nettle D, et al. (2013) The watching eyes effect in the Dictator Game: It’s not how much you give, it’s being seen to give something. *Evol Hum Behav* 34(1):35–40.

80. Noussair C, Tucker S (2007) Public Observability of Decisions and Voluntary Contributions in a Multiperiod Context. *Public Financ Rev* 35(2):176–198.

81. Oda R, Ichihashi R (2016) Effects of Eye Images and Norm Cues on Charitable Donation: A Field Experiment in an Izakaya. *Evol Psychol* 14(4):1–6.

82. Oda R, Niwa Y, Honma A, Hiraishi K (2011) An eye-like painting enhances the expectation of a good reputation. *Evol Hum Behav* 32(3):166–171.

83. Panagopoulos C (2014) Watchful eyes: Implicit observability cues and voting. *Evol Hum Behav* 35(4):279–284.

84. Panagopoulos C (2014) I’ve Got My Eyes on You: Implicit Social-Pressure Cues and Prosocial Behavior. *Polit Psychol* 35(1):23–33.

85. Panagopoulos C (2010) Affect, Social Pressure and Prosocial Motivation: Field Experimental Evidence of the Mobilizing Effects of Pride, Shame and Publicizing Voting Behavior. *Polit Behav* 32(3):369–386.

86. Pedersen R (2016) *No Effects of Artificial Surveillance Cues or Social Proofs on Survey Participation Rates*.

87. Pfattheicher S, Keller J (2015) The watching eyes phenomenon: The role of a sense of being seen and public self-awareness. *Eur J Soc Psychol* 45:560–566.

88. Pfattheicher S (2015) A regulatory focus perspective on reputational concerns: The impact of prevention-focused self-regulation. *Motiv Emot* 39(6):932–942.

89. Piazza J, Bering JM (2008) Concerns about reputation via gossip promote generous allocations in an economic game. *Evol Hum Behav* 29(3):172–178.

90. Powell KL, Roberts G, Nettle D (2012) Eye Images Increase Charitable Donations: Evidence From an Opportunistic Field Experiment in a Supermarket. *Ethology* 118(11):1096–1101.

91. Raihani NJ, Bshary R (2012) A positive effect of flowers rather than eye images in a large-scale, cross-cultural dictator game. *Proc Biol Sci* 279(1742):3556–64.

92. Reinstein D, Riener G (2012) Reputation and influence in charitable giving: An experiment. *Theory Decis* 72(2):221–243.

93. Rigdon M, Ishii K, Watabe M, Kitayama S (2009) Minimal social cues in the dictator game. *J Econ Psychol* 30(3):358–367.

94. Roth AE (1995) Bargaining Experiments. *The Handbook of Experimental Economics*, eds Hagel JH, Roth AE (Princeton University Press, Chichester), pp 292–304.

95. Samek A, Sheremeta RM, Angeles L (2015) Selective Recognition : How to Recognize Donors to Increase Charitable Giving. 1–19.

96. Samek A, Sheremeta RM (2016) When Identifying Contributors is Costly: An Experiment on Public Goods. *South Econ J* 82(3):801–808.

97. Samek A, Sheremeta RM (2014) Recognizing contributors: an experiment on public goods. *Exp Econ*:1–18.

98. Satow KL (1975) Social approval and helping. *J Exp Soc Psychol* 11(6):501–509.

99. Saunders TJ, Taylor AH, Atkinson QD (2016) No evidence that a range of artificial monitoring cues influence online donations to charity in an MTurk sample. *R Soc Open Sci* 3(10). doi:10.1098/rsos.150710.

100. Schmidt C, Zultan R (2005) The uncontrolled social utility hypothesis revisited. *Econ Bull* 3(1):1–8.

101. Sénémeaud C, et al. (2017) The watching-eyes phenomenon and blood donation: Does exposure to pictures of eyes increase blood donation by young adults? *Transfus Apher Sci*:2016–2018.

102. Smith FG, et al. (2009) Attractiveness qualifies the effect of observation on trusting behavior in an economic game. *Evol Hum Behav* 30(6):393–397.

103. Sparks A, Barclay P (2013) Eye images increase generosity, but not for long: The limited effect of a false cue. *Evol Hum Behav* 34(5):317–322.

104. Sparks A (2010) Subtle Cues and Economic Games.

105. Tane K, Takezawa M (2011) Perception of human face does not induce cooperation in darkness. *Lett Evol Behav Sci* 2(2):24–27.

106. Thielmann I, Heck DW, Hilbig BE (2016) Anonymity and incentives: An investigation of techniques to reduce socially desirable responding in the Trust Game. *Judgm Decis Mak* 11(5):527–536.

107. Uziel L, Hefetz URI (2014) The Selfish Side of Self-Control. 458(August):449–458.

108. van Bommel M, van Prooijen JW, Elffers H, van Lange P a. M (2014) Intervene to be Seen: The Power of a Camera in Attenuating the Bystander Effect. *Soc Psychol Personal Sci* 5(4):459–466.

109. van Rompay TJL, Vonk DJ, Fransen ML (2009) The Eye of the Camera. Effects of security Cameras on Prosocial Behavior. *Environ Behav* 41(1):60–74.

110. van Vugt M, Hardy CL (2010) Cooperation for reputation: Wasteful contributions as costly signals in public goods. *Gr Process Intergr Relations* 13(1):101–111.

111. Van Vugt M, Iredale W (2013) Men behaving nicely: Public goods as peacock tails. *Br J Psychol* 104(1):3–13.

112. Vogt S, Efferson C, Berger J, Fehr E (2015) Eye spots do not increase altruism in children. *Evol Hum Behav* 36(3):224–231.

113. Wichman H (1970) Effects of isolation and communication on cooperation in a two-person game. *J Pers Soc Psychol* 16(1):114–120.

114. Vrouwe W (2014) Helping a child: the watching eyes effect further examined. Available at: https://www.kl.nl/wp-content/uploads/2015/03/MT_Helping_Child_WEF_Further_Examined_WV.pdf.

115. Wilson RK, Sell J (1997) “Liar, Liar... ” Cheap Talk and Reputation in Repeated Public Goods Settings. *J Conflict Resolut* 41(5):695–717.

116. Xin Z, Liu Y, Yang Z, Zhang H (2016) Effects of minimal social cues on trust in the investment game. *Asian J Soc Psychol* 19(3):235–243.

117. Yoeli E, Hoffman M, Rand DG, Nowak MA (2013) Powering up with indirect reciprocity in a large-scale field experiment. *Proc Natl Acad Sci U S A* 110(Supplement 2):10424–9.

Supplementary File S2 Publication Bias

The symmetry within the funnel plot (see Figure S1) indicates that there is no publication bias, as well as, the non-significant Egger’s regression test (*p* = .153). In addition, Duval and Tweedie's (2000) trim and fill procedure indicated no ‘missing’ studies were below the average effect size suggesting publication bias has not artificially inflated the effect size.


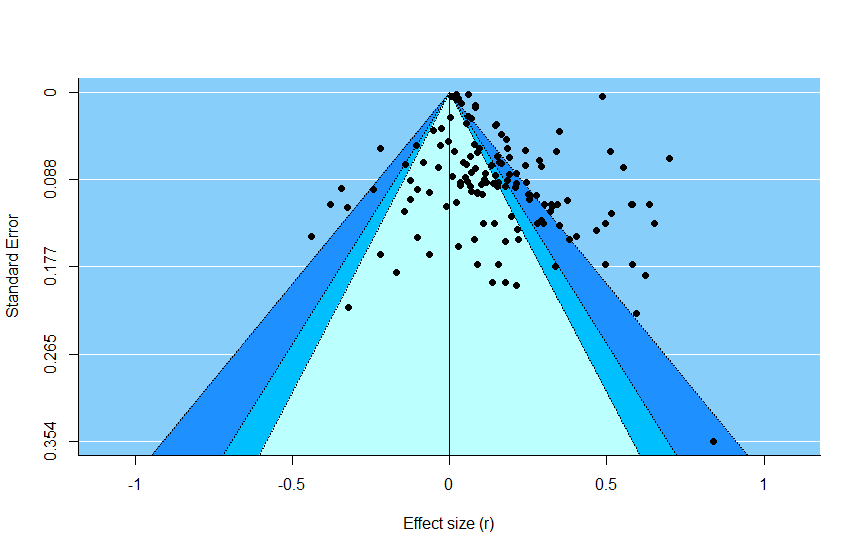


Figure S2. Funnel Plot of effect sizes by the standard error.

Supplementary File S3 Univariate Moderator Analyses

Table S3 displays the number of studies, sample size, heterogeneity statistics and effect size of all the univariate moderators.

Table S3. Univariate moderator analysis on the relationship between observability and prosocial behaviour

|  | QM | p value (two tailed) | *K* | N | Tau2 | Standard Error | r | 95% LLCI/ULCI |
| --- | --- | --- | --- | --- | --- | --- | --- | --- |
| Type of Observability | 3.575 | .167 |  |  |  |  |  |  |
| Perceived |  |  | 49 | 158,166 | 0.023 | 0.025 | 0.100 | 0.050/0.149 |
| Pseudo Observable |  |  | 20 | 3,876 | 0.028 | 0.044 | 0.167 | 0.081/0.252 |
| Overt Observable |  |  | 60 | 625,508 | 0.044 | 0.030 | 0.172 | 0.113/0.232 |
| Type of Observer | 13.246 | 0.004** |  |  |  |  |  |  |
| None |  |  | 49 | 158,166 | 0.023 | 0.025 | 0.100 | 0.050/0.149 |
| Experimenter |  |  | 12 | 362,668 | 0.028 | 0.056 | 0.200 | 0.090/0.309 |
| Peers |  |  | 45 | 8,158 | 0.046 | 0.035 | 0.137 | 0.085/0.224 |
| Passive Observers |  |  | 12 | 970 | 0.029 | 0.064 | 0.352 | 0.225/0.478 |
| Decision with Consequences | 7.365 | 0.007** |  |  |  |  |  |  |
| Consequence free |  |  | 110 | 785,244 | 0.026 | 0.018 | 0.119 | 0.084/0.154 |
| Decisions with consequences |  |  | 24 | 2,920 | 0.048 | 0.051 | 0.248 | 0.137/0.347 |
| Single/Repeated | 12.443 | <0.001** |  |  |  |  |  |  |
| Single |  |  | 94 | 781,352 | 0.027 | 0.005 | 0.106 | 0.070/0.143 |
| Repeated |  |  | 25 | 3,026 | 0.037 | 0.046 | 0.269 | 0.179/0.358 |
| Barging/Social Games | 6.963 | 0.008*** |  |  |  |  |  |  |
| Bargaining games |  |  | 74 | 20,968 | 0.030 | 0.024 | 0.119 | 0.072/0.165 |
| Social Dilemma games |  |  | 26 | 2,541 | 0.046 | 0.048 | 0.251 | 0.157/0.334 |
| Methodological |  |  |  |  |  |  |  |  |
| Age | 0.219 | 0.639 | 65 | 347,981 | 0.021 | 0.002 | -0.001 | -0.005/0.003 |
| Type of Payment | 1.896 | 0.388 |  |  |  |  |  |  |
| No Payment |  |  | 18 | 11,449 | 0.009 | 0.004 | 0.139 | 0.085/0.194 |
| One off payment |  |  | 18 | 1,882 | 0.035 | 0.051 | 0.080 | -0.021/0.181 |
| Performance Related payment |  |  | 80 | 12,479 | 0.042 | 0.026 | 0.157 | 0.106/0.208 |
| Context of Study | 6.215 | 0.013* |  |  |  |  |  |  |
| Lab |  |  | 99 | 12,001 | 0.039 | 0.023 | 0.170 | 0.125/0.215 |
| Non Lab |  |  | 35 | 776,163 | 0.015 | 0.023 | 0.077 | 0.033/0.121 |
|  |  |  |  |  |  |  |  |  |
|  |  |  |  |  |  |  |  |  |
|  | **QM** | **p value (two tailed)** | ***K*** | **N** | **Tau2** | **Standard Error** | **r** | **95% LLCI/ULCI** |
|  |  |  |  |  |  |  |  |  |
| Blind/Double Blind | 0.955 | .328 |  |  |  |  |  |  |
| Blind |  |  | 106 | 718,953 | 0.029 | 0.019 | 0.130 | 0.092/0.168 |
| Double Blind |  |  | 18 | 65,643 | 0.030 | 0.011 | 0.178 | 0.087/0.268 |
| Nature of Outcome | 0.419 | .517 |  |  |  |  |  |  |
| Whether to help |  |  | 29 | 709,212 | 0.026 | 0.033 | 0.131 | 0.066/ 0.196 |
| How much to help |  |  | 78 | 71,640 | 0.035 | 0.024 | 0.161 | 0.113/0.209 |
| Unearned vs Earned | 1.134 | 0.241 |  |  |  |  |  |  |
| Unearned |  |  | 91 | 14,416 | 0.039 | 0.024 | 0.133 | 0.087/0.180 |
| Earned |  |  | 10 | 9,883 | 0.054 | 0.080 | 0.222 | -0.065/0.379 |
| Measure of Outcome | 1.130 | 0.288 |  |  |  |  |  |  |
| Objective |  |  | 122 | 784,237 | 0.033 | 0.019 | 0.146 | 0.109/0.18 |
| Subjective |  |  | 10 | 3,820 | 0.016 | 0.047 | 0.081 | -0.100.172 |
| Type of Prosocial Behavior | 0.686 | 0.408 |  |  |  |  |  |  |
| Monetary |  |  | 101 | 77,267 | 0.039 | 0.022 | 0.151 | 0.107/0.195 |
| Non Monetary |  |  | 33 | 710,897 | 0.016 | 0.005 | 0.115 | 0.067/0.163 |
| Aggregated Data | 3.597 | 0.058 |  |  |  |  |  |  |
| Not Aggregated |  |  | 129 | 734,978 | 0.028 | 0.017 | 0.133 | 0.099/0.166 |
| Aggregated |  |  | 5 | 53,186 | 0.066 | 0.044 | 0.290 | 0.059/0.521 |

Supplementary File S4 Meta Regression Table.

Table S4 shows the effect of the significant univariate moderators on the link between observability and prosocial behaviour. No moderators except for single/repeated measures were significant in the meta-regression.

Table S4. *Meta-Regression.*

|  | B (SE) | 95% Ci LL/UP |
| --- | --- | --- |
| Intercept | 0.103(0.040) | 0.025/0.181 ** |
| Type of Observer (Base = None) |  |  |
| Experimenter | 0.114(0.075) | -0.034/0.261 |
| Peers | 0.040 (0.062) | -0.082/0.162 |
| Passive Observers | .171 (0.092) | -0.009/0.352 |
| Decisions with Consequences | -0.024 (0.076) | -0.174/0.125 |
| Context of Study (lab = 0, No lab =1) | -0.098 (0.061) | -0.217/0.022 |
| Single/Repeated (Base = Single) | 0.140 (0.070) | 0.003/0.276 * |
| Bargaining (0) or Social Dilemma games (1) | 0.017 (0.071) | -0.123/0.157 |
| R² | 9.26% |  |

Note 1. * p <. 05. Model: QM (7) = 17.02, p = .017; QE (77) = 387.515, p < .001. k = 85. * .05, ** .01, ***.001. Results remain the same if the aggregated data moderator is removed from the model.

Supplementary File S5 PRISMA Flow Diagram

Figure S5 shows the flow of studies included within the meta-analysis.

Search of Electronic Databases yielded: 11,339 were searched through.

Number of full-text articles/ book chapters/ working papers/ doctoral theses/ Unpublished study data assessed for after initial screening (through abstracts or titles): 114

Review of titles and abstract yielded 91 papers

Number of articles included in the present analysis: 117

Number of studies included in the present analysis: 134

Backward searching led to a further 55 records being identified. Giving a total of 169 full-text articles/ book chapters/ working papers/ doctoral theses/ unpublished study data.

Email to unique first authors (61) requesting unpublished data led to an additional 18 record being identified. A reviewer recommended a further 15 records. Giving a total of 202 full-text articles/ book chapters/ working papers/ doctoral theses/ unpublished study data.

Number of deleted entries due to duplicating data sets: 7

Number of records ruled out after initial screening: 9,964

Number of deleted entries out of lack of data: 2

Number of deleted upon further scrutiny of design and measures: 67

Number of deleted entries due to providing no primary data: 9

*Figure S5*. PRISMA flow diagram illustrating the inclusion and exclusion of studies during the literature search.

Supplementary File S6 Coding Scheme

Coding Scheme

See Table S6 for full details of the coding scheme.

Theoretical Moderators.

Type of Observability. We conceptualised observability manipulations as falling into one of three categories: the ‘sense of being watched’ (perceived observability; k = 49), a unique identity reference that is viewed by others (pseudo observability; k = 20), and personal information (i.e. name, picture of face etc.; k = 60) viewed by others (overt observability).

***Observer Type.*** We investigated whether the type of observer influences the strength of the relationship between observability and prosociality. Observer type was coded into 4 categories: no-one watching (*k* = 49), experimenter observing behaviour (*k* = 12), peers watching behaviour (*k* = 45), and passive observer (an individual who is observing and is not a participant or experimenter) (*k* = 12).

***Decisions with Consequences.*** We coded whether the effect size in the observable conditions will influence how others (experimenter, peers or independent passive observer) respond towards the behaviour of the person being observed (the allocator). Decisions with consequences could be in the form of either a behavioural response or explicit evaluation (*k* = 24). Consequence-free decisions occur (when the participant is aware that the observer cannot actively influence the participant) (*k* = 110).

***Single or repeated measures.*** We coded whether the experiment was a single game including studies where the same single game is played with different people (stationary replication, see Camerer and Fehr, 2002)(*k* = 94), or repeated measures (same game with the same people) (*k* = 25).

***Bargaining games and social dilemmas.*** We coded for the type of economic game used: bargaining games (*k* = 74) and social dilemmas (*k* = 26). In bargaining games, agents decide how to divide some amount (e.g. Dictator Game) and in social dilemma games, individuals profit from being selfish unless everyone chooses the selfish option at which point everyone loses (e.g. public goods games).

Methodological Moderators.

***Age.*** We recorded the mean ages of participants when this was reported (Mean = 24.95, SD = 10.21, *k* = 65).

***Type of payment.*** To explore if the method of payment effected prosocial behaviour a moderator for the type of payment was coded which contained 3 categories: No payment (Base level; *k* = 18), one-off payment (*k* = 18), performance related payment (*k* = 80).

***Context of Study.*** We coded whether studies were performed in a laboratory (*k* = 99) or field setting (total *k* = 35; online = 7, field = 28)*.*

***Single vs Double Blind.*** We assessed whether single or double-blind procedures were applied: coded as single-blind (*k* = 106), or double-blind (*k* = 18).

***Nature of the outcome.*** We coded whether the researchers examined behaviour in terms of whether to help or donate (*k* = 29) or how much to help or donate (*k* = 78).

***Unearned vs earned.*** Studies were coded to explore whether simply giving participants money (*k* = 91) or having participants earn their money (*k* = 10) moderated the observability - prosocial behaviour link.

***Measures of outcome.*** The measure of prosocial behaviour was coded as either objective (behavioural: *k* = 122), or subjective (self-reported or rated by a third party; *k* = 10).

***Type of Prosocial Behaviour.*** We examined if the effect of visibility on prosocial behaviour was the same when the decision involved monetary (*k* = 101) or non-monetary (*k* = 33) responses.

***Aggregated data.*** A number of studies only reported aggregated effects, therefore this was included as a potential moderator: Aggregated yes (*k* = 5) or no (*k* = 125).

Table S6.

Definition of Key Moderators

| Moderator | Working Definition and codes | Examples from Included samples |
| --- | --- | --- |
| Theoretical Moderators |  |  |
| Type of Observability Manipulation (*k =* 129 | Observability manipulations were split into three: Perceived, Pseudo and Overt.  Perceived observability induces the ‘feeling of being watched’ i.e. displaying eyes or priming observability (*k* = 49).  Pseudo observable is where information that indirectly (e.g., ID number) reveals participants identity is visible (*k* = 20).  Overt observability is where information directly related to participants identity (i.e. pictures, names, voices etc.) are revealed (*k* = 60). | An example of perceived observability: Haley and Fessler, (2005) displayed an image of eyes on the background of a pc whilst participants made donation decisions.  An example of Pseudo observability: Wilson and Sell, (1997) assign an ID number to each participant that is then displayed to other players with their contributions amount.  An example of overt observability: Samek and Sheremeta, (2014) in a public goods game displayed in the visible conditions name, picture and amount donated by each allocator. |
| Type of Observer. (*k =* 118) | No one is watching(0) (*k* = 49) (i.e Perceived Observability manipulations), the experimenter can observer behaviour (1) (*k* = 12), peers involved in the experiment are aware of the allocators behaviour (2) (*k* = 45), a passive observer who is someone who is not participating in the study or involved in running the experiment instead they are usually just observing behaviour with no ability to punish or recompense others (3) (*k* = 12), | Haley and Fessler, (2005) no one is actually watching (0). Hoffman, McCabe, Shachat, and Smith, (1994) looks at whether the experimenter knowing about participants response effects influences behaviour (single vs double blind)(1).  Bereczkei, Birkas, and Kerekes, (2007) offers of help in front of their seminar group(2). Izuma, Saito, and Sadato, (2010) had performance on a donation task observed by two actors (who were not experiments)(3). Alevy, Jeffries, and Lu, (2014) displayed information on the whiteboard so it was visible to other players and the experimental monitor(4). |
| Moderator | **Working Definition and codes** | **Examples from Included samples** |
| Decisions with consequences vs Consequence free decisions  (*k =* 134) | Consequence-free decisions refer to allocation decisions that are standalone and cannot impact how others respond to the allocator within the experimental protocol (0) (*k* = 110). Decisions with consequences refer to allocation decisions that could impact how others (fellow participants, experimenters or passive observers) respond to the allocator either in terms of behavioural responses or explicit evaluations within the experimental protocol (1) (*k* = 24). | Izuma et al., (2010) had participants in the visible condition that are told that the observers are completing an impression formation task about them whilst they complete a charity dictator game task. |
|  |  |  |
| Methodological Moderators |  |  |
| Type of payment  (*k =* 116) | No payment (0) (*k =* 18), One-off payment (i. e. show up fee,) (1) (*k =* 18). Performance related payment (where earning vary on how participant do) (2) (*k =*  80) (if study has both show up fee and performance it is classified as performance) | Jerdee and Rosen, (1974) participants’ were enrolled on business administration course and as such were not paid to take part (0)Carbon and Hesslinger, (2011) participants’ were given course credits for taking part in the study (1). Kurzban, (2001) participants were paid $2 for turning up on time and 50 cents for every token they earned on average over the course of the 10 ten rounds in the public goods game (2). |
| Context of Study: Online, lab and field  (*k =* 134) | Was the study conducted in the laboratory (0) (*k* = 99) or non-laboratory (field and online) (*k* = 35) | Pfattheicher, (2015) study 2 sample 2 used 123 participants recruited via amazon mechanical Turk and conducted online (1). Matland and Murray, (2015) look at the effect of eyes on American voters across a number of states (field study)(2). Hardy and Van Vugt, (2006) contains two relevant experiments both of which occur within the laboratory (3). |
| Moderator | **Working Definition and codes** | **Examples from Included samples** |
| Single or Repeated Measures  (*k =* 119) | Single measure is a situation where the allocation decision is made once with the same group of people or there are multiple one-off allocations with different people (i.e. stationary replications) (0) (*k =* 94). For repeated measures, the allocation decision must be made more than once with the same group of people (1) (*k =* 25). | Rege and Telle, (2004) use a public goods game with multiple session. In session 1-4 interactions are single (0) whilst in session, 4-12 interaction is repeated over 20 rounds (1). |
| Blind  (*k =* 124) | Was the design single blind (1) (*k* = 106), or double blind (2) (*k* = 18). |  |
| Bargaining games or Social Dilemma’s  (*k =* 107) | Whether the economic game is a Bargaining Game (i.e., Dictator Game, Ultimatum game, Trust game etc) (*k =* 74) vs a Social Dilemma (i.e. Public goods games, Prisoner Dilemma or limited resource allocation game etc.) (*k =* 26). | Fehr and Schneider, (2010) one-shot trust game is an example of a bargaining game (0) whereas, Samek and Sheremeta, (2016)public goods game is an example of a social dilemma scenario (1). |
| Nature of Outcome: Helped and how much (*k =* 107) | Whether or not participant helps (*k* = 29) and if so how much the participants helped (*k* = 78). | Nettle et al., (2013) looked at whether the watching eyes effect influenced more whether people decide to donate as opposed to how much participants decide to donate. |
| Unearned or Earned Endowment  (*k =* 101) | Whether the endowment was earned during the experiment (1) (*k =* 10) or freely given by the experimenter (0) (*k =* 91). | Nettle et al.,(2013) left £5 on a desk for participants (0). Satow, (1975) had participant earn money by answering word puzzles (1). |
| Measure of Outcome: Objective vs subjective  (*k =* 132) | Objective (behavioural) (1) (*k* = 122) vs subjective (Self report and third party) (*k* = 10) (0) | Lambarraa and Riener, (2015). The outcome measure was the amount of money participants choose to keep or donate (1). Bereczkei, Birkas, and Kerekes, (2010) asked participants to self-report which charitable activities they would be willing to help with (0).van Rompay, Vonk, & Fransen, (2009) has experimenters, blind to the hypothesis, code participants helping behaviour after experimenter had dropped a pile of surveys (0). |
| Type of Prosocial Behaviour: Money vs other  (*k = 134*) | Monetary (*k =* 101 ) (0) or Non-Monetary (e.g., effort, time) (*k =* 33 )(1). | Receiving payment from a lab task (0) vs giving blood, voting or exert effort(1)(8, 14, 107, 108). |
| Aggregated Data  (*k =* 134) | Was the effect size generated from individual or group level data?  Yes (*k* = 5) or No (*k* = 129) response. | Powell, Roberts, and Nettle, (2012) reported results per 000’s customers (1). |

Supplementary File S7 Overview of Analysis.

**Using a Random Effects Model.** A random effects model was used to compute the overall effect size of observability on prosociality. A random effects model was chosen a) because we are interested in making generalizable inferences about the effect, and b) given the variability in effects sizes between observability and pro-sociality it is unlikely that there is a single common effect hence assuming a distribution of effects is more appropriate (1, 2).

**Independence of Effects**. Many of the studies included in the meta-analysis had multiple effect sizes either due to multiple trial arms on one dependent variable or using the same dependent variable twice (e.g. in the “watching eyes” literature, the same dependent variable is used to assess *whether* a participant gives and *how much* they give) (e.g. Raihani and Bshary, 2012). Effect sizes that arise from the same study are not independent (pg. 191-195 Card, 2012) and can lead to an upward bias of population and study level variance estimates (see pg. 432, Hunter & Schmidt, 2004).

**Assessing Publication Bias**. Publication bias was assessed visually with a funnel plot (effect size plotted against the standard error) and statistically using Egger’s Regression test (6). Eggers regression test uses the effect size as the DV and the precision (inverse of the studies standard error) as the predictor in a simple regression model. If the intercept is significant, this means that small studies have different effects than large studies, suggesting publication bias. The Duval and Tweedie, (2000) trim and fill procedure was also applied. This approach works by assuming that symmetry within a funnel plot implies no bias. The trim and fill procedure trims extreme values and then fills in estimated values. This process is iterative and stops once symmetry has been achieved. The trim and fill procedure gives an adjusted estimate of the effect size and indicates the number of potential missing studies.

**Identifying Heterogeneity.** Cochran’s Q test, a measure of homogeneity among effect sizes, was conducted to test whether the assumption that all effect sizes are estimating the same population mean is reasonable. If this test is significant it means that variability across effect sizes is more than would be expected by sampling error alone (58). The interpretation of this test changes in mixed effects models where the observed variability in effect size, not accounted for by moderators, is larger than would be expected based on sampling variance alone (reported as *QE*). To measure the degree of heterogeneity, the inconsistency index (*I*²) and tau squared (τ²) were calculated. The inconsistency index indicates (as a percentage) how much of the total variability (heterogeneity plus sampling error) in effect sizes can be attributed to heterogeneity between the true effect sizes. For mixed effects, the interpretation changes slightly to how much of the unaccounted variability (made up of residual and sampling heterogeneity) can be explained by residual heterogeneity (59). Thus, large inconsistency index percentages in random effects models indicate that more of the variability is explained by differences among the true effect size, and in mixed effects models as more residual heterogeneity. Tau squared represents the amount of residual heterogeneity in the random/mixed effects models with larger tau squares indicating more residual heterogeneity.

Coding frame reliability

The first and third authors created the initial coding framework. The first author coded all the studies. The first and third author discussed ambiguous cases and agreed on the final coding. The reliability of the coding framework was tested on a random sample of 32% of studies (*k* = 43) and a third rater (blind to initial coding) applied the framework. The tests of the reliability of the coding frame were applied to all codes except those that were objectively attained (i.e. unearned vs earned, Age, type of payment, context of study, bargaining games and social dilemmas, nature of the outcome, measure of the outcome, and aggregated data). The kappa coefficients indicate substantial or greater agreement (mean *Kappa* = .86, SD = . 13, min = .67, max = 1.00)(8).
